# Yogurt Benefits Bone Mineralization in Ovariectomized Rats with Concomitant Modulation of the Gut Microbiome

**DOI:** 10.1002/mnfr.202200174

**Published:** 2022-09-15

**Authors:** Weiwei He, Zhuqing Xie, Nina Kølln Wittig, Line F. Zachariassen, Amanda Andersen, Henrik J. Andersen, Henrik Birkedal, Dennis S. Nielsen, Axel K. Hansen, Hanne Christine Bertram

**Affiliations:** ^1^ Department of Food Science Aarhus University Agro Food Park 48 Aarhus N 8200 Denmark; ^2^ Department of Food Science University of Copenhagen DK‐1958 Frederiksberg Denmark; ^3^ Department of Chemistry and iNANO Aarhus University DK‐8000 Aarhus C Denmark; ^4^ Department of Veterinary and Animal Sciences Faculty of Health and Medical Sciences University of Copenhagen DK‐1958 Frederiksberg Denmark; ^5^ Arla Food Ingredients Sonderhoj 10 Viby DK‐8260 Denmark; ^6^ Arla Food amba Agro Food Park 19 Aarhus DK‐8200 Denmark

**Keywords:** fermented dairy, gut metabolome, gut–bone axis, inulin, NMR metabolomics

## Abstract

**Scope:**

Evidence supports that gut‐modulating foods potentially can suppress bone loss in postmenopausal women. This study aims to investigate the effect of milk calcium‐enriched milk, yogurt, and yogurt‐inulin combination on the gut–bone association.

**Methods and results:**

A 6‐week intervention study is conducted in ovariectomized rats. Four pastes containing milk calcium‐fortified milk (M‐Ca), milk calcium‐fortified yogurt (Y‐Ca), inulin‐fortified Y‐Ca (Y‐I‐Ca), or an isoconcentration of calcium carbonate (Ca‐N), and a calcium‐deficient paste are provided. M‐Ca does not influence bone mineral density and content (BMD and BMC), femur mechanical strength, or femoral microstructure compared to Ca‐N, but Y‐Ca increases spine BMD. The serum metabolome reveals that Y‐Ca modulated glycine‐related pathways with reduced glycine, serine, and threonine. No additive effects of yogurt and inulin are found on bone parameters. Correlation analysis shows that increased lactobacilli and reduced *Clostridiaceae* members in Y‐Ca is associated with an increased spine BMD. Increases in *Bifidobacterium pseudolongum*, *Turicibacter*, *Blautia*, and *Allobaculum* and gut short‐chain fatty acids in Y‐I‐Ca are not reflected in bone parameters.

**Conclusion:**

Yogurt as calcium vehicle contributes to increased spine BMD concomitant with changes in the gut microbiome and glycine‐related pathways, while adding inulin to yogurt does not affect bone mineralization in ovariectomized rats.

## Graphical abstract

This study investigates how milk, yogurt, and yogurt‐inulin combination influence bone mineralization in ovariectomized rats. Results indicate that a beneficial effect of yogurt on bone mineralization involves increased lactobacilli, reduced *Clostridiaceae* gut microbiota members, and simultaneous changes in glycine‐related metabolism. Neither milk nor addition of inulin to yogurt affect the measured bone mineralization, although inulin supplementation markedly modulates gut environment.

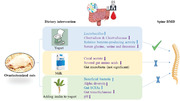

## 1 Introduction

Osteoporosis is a systemic skeletal disease characterized by a reduced bone mineral density (BMD) which exposes elderly to a high risk of fracture.^[^ ^1^ ^]^ Intriguingly, osteoporosis in women is twice as likely as men after the age of 50 years.^[^ ^2^ ^]^ Estrogen plays an important role in the inhibition of bone remodeling, which explains why women experience a rapid bone loss in the early stages of menopause (decline in estrogen de novo synthesis).^[^ ^1^ ^]^ Menopause has recently been reported to be accompanied by changes in the gut microbiome composition and function,^[^ ^3^, ^4^ ^]^ and a potential relationship between gut microbiome and bone metabolism has therefore received attention.^[^ ^5^ ^]^ To investigate the role of gut microbiome in estrogen deficiency, Li et al.^[^ ^6^ ^]^ implemented a murine model and found that ovariectomy‐induced bone loss is lower in germ‐free mice than in conventional mice. Furthermore, ovariectomy‐induced bone loss in conventional mice could be attenuated by treatment with probiotics, including *Lactobacillus* *rhamnosus* GG and the commercial probiotic mixture VSL#3 (containing eight bacterial strains).^[^ ^6^ ^]^ Hypotheses explaining how the modification of gut microbiome impacts bone metabolism involve a reduction in bacteria‐induced inflammatory response on osteoclast‐mediated bone resorption^[^ ^7^, ^8^ ^]^ and effects on bacterial‐derived metabolites (e.g., short‐chain fatty acids (SCFAs)) involved in the regulation of calcium absorption.^[^ ^9^ ^]^

Emerging evidence suggests that intake of fermented dairy such as yogurt is associated with an increased BMD and a reduced risk of fracture in postmenopausal women.^[^ ^10^, ^11^ ^]^ Besides being rich in calcium and milk proteins,^[^ ^12^ ^]^ fermented dairy products also contain starter cultures. Interestingly, in adults with low‐grade inflammation and high risk of osteoporosis, intake of yogurt increased the abundance of some species of *Lactobacillus* and *Streptococcus* that are commonly used starters in yogurts and simultaneously increased bone formation markers.^[^ ^13^ ^]^ Moreover, a double‐blinded randomized control study in postmenopausal women showed that intake of three different *Lactobacillus* strains over a period of 7 months reduced bone loss significantly.^[^ ^14^ ^]^ Changes in gut microbiota upon yogurt consumption also alter the gut metabolome, e.g., propanoate and butanoate production with potential to maintain systemic homeostasis, such as gut barrier function.^[^ ^15^ ^]^ However, whether certain gut‐derived metabolites are causal links between yogurt consumption and bone health remain unknown. Consequently, discovery of related causes are important for developing dietary strategies to promote bone health.

Prebiotics (e.g., inulin and resistant starch^[^ ^16^ ^]^), may be added to yogurt as synergistically “bone‐friendly” nutrients as a result of their ability to stimulate growth and activity of certain gut microbes and hereby mutually lower colon pH^[^ ^13^, ^17^ ^]^ and stimulate SCFAs production—both of which have been found to impact bone metabolism.^[^ ^9^, ^18^ ^]^

To investigate the effects of dairy matrixes (milk calcium‐enriched milk and milk calcium‐enriched yogurt) and addition of inulin to yogurt on bone mineralization, a 6‐week dietary intervention study was conducted in ovariectomized (OVX) rats to simulate the physiological condition of estrogen deficiency in postmenopausal women. In addition, to provide further evidence for the proposed gut–bone axis, the present study also elucidated the potential associations between bone mineralization and specific gut bacteria, as well as the gut and serum metabolomes.

## 2 Results

### 2.1 Diet Intervention and Bone parameters

During the intervention, all rats had a significant BW gain (Figure S1, Supporting Information). Rats fed Y‐Ca experienced a higher weight gain compared to the other four groups, but BW differences at the end of intervention were not significant.

The total BMD, total BMC, spine BMD, and spine BMC measured by DXA are shown in **Table** **1**. Rats fed the calcium‐deficient diet (Ca‐D) had significantly lower BMD and BMC compared with the four calcium‐fortified diets (*p* < 0.05). Rats fed Y‐Ca had a higher spine BMD than the Ca‐N group (*p* < 0.05) and a higher spine BMC than the M‐Ca group and the Ca‐N group (*p* < 0.05). In addition, rats fed Y‐I‐Ca tended to have a slightly higher spine BMD than the Ca‐N group even not statistically significant (*p* = 0.096). To adjust for BW variations (Figure S2, Supporting Information), BMC was normalized to BW to display the relative BMC. Although there were no significant differences in the relative total and spine BMC among the four calcium‐fortified groups, a slightly higher relative spine BMC was observed in rats fed Y‐Ca and Y‐I‐Ca in comparison to the M‐Ca group and the Ca‐N group.

**Table 1:** Bone parameters (mean ± SD) of OVX rats fed the different diets

Bone parameters	Ca‐D	Ca‐N	M‐Ca	Y‐Ca	Y‐I‐Ca	*p* value
BMD and BMC (*n* = 44)						
Total BMD [g cm^─2^]	0.157 ± 0.008	0.168 ± 0.010^*^	0.173 ± 0.006^*^	0.176 ± 0.014^*^	0.175 ± 0.013^*^	0.006
Spine BMD [g cm^─2^]	0.140 ± 0.005	0.160 ± 0.007^*^	0.164 ± 0.009^*^	0.176 ± 0.029^*#^	0.173 ± 0.007^*^	<0.001
Total BMC [g]	6.97 ± 1.48	8.59 ± 1.14^*^	8.46 ± 0.58^*^	9.33 ± 1.52^*^	8.18 ± 0.87^*^	0.004
Spine BMC [g]	1.25 ± 0.28	1.63 ± 0.19^*^	1.56 ± 0.17^*^	1.90 ± 0.44^*#^	1.68 ± 0.22^*^	<0.001
Relative total BMC [g kg^─1^]	20.9 ± 3.2	25.2 ± 3.1^*^	24.3 ± 1.8^*^	24.5 ± 2.3^*^	24.2 ± 2.4^*^	0.018
Relative spine BMC [g kg^─1^]	3.76 ± 0.79	4.78 ± 0.55^*^	4.54 ± 0.55	5.02 ± 1.12^*^	4.99 ± 0.90^*^	0.019
Mechanical femur strength (*n* = 44)						
Max. load [N]	93.9 ± 5.5	109.7 ± 6.5^*^	105.7 ± 9.7^*^	109.1 ± 11.2^*^	108.3 ± 14.8^*^	0.017
Femur structure (*n* = 25)^¤^						
Tb.BV [mm^3^]	1.91 ± 0.37	2.59 ± 0.50	2.51 ± 1.30	3.36 ± 1.82	3.39 ± 1.40	0.14
Tb.BV/TV	0.081 ± 0.022	0.125 ± 0.021	0.130 ± 0.086	0.145 ± 0.053	0.145 ± 0.040	0.078
Tb.Th [µm]	72.7 ± 1.9	81.5 ± 2.8^*^	80.1 ± 2.6^*^	83.5 ± 3.7^*^	84.2 ± 5.9^*^	<0.001
Tb.Sp [mm]	1.14 ± 0.31	0.90 ± 0.21	0.91 ± 0.40	0.80 ± 0.21	0.82 ± 0.29	0.41
Ct.BV [mm^3^]	5.25 ± 0.24	5.90 ± 0.36	5.92 ± 0.53	5.69 ± 0.20	6.09 ± 0.49	0.052
Ct.BV/TV	0.39 ± 0.06	0.46 ± 0.03	0.46 ± 0.04	0.44 ± 0.07	0.45 ± 0.05	0.59
Ct.Th [µm]	157 ± 26	159 ± 6	171 ± 21	161 ± 16	169 ± 5	0.22

¤Five femurs in each group were assessed by X‐ray computed tomography. BV, bone volume; Ct: cortical; Sp: separation; Tb: trabecular; Th, thickness; TV, tissue volume.

*p < 0.05 compared to Ca‐D. ^#^ *p* < 0.05 compared to Ca‐N.

For the femur bone, calcium supplementation significantly increased the mechanical bone strength as evaluated by three‐point bending tests as well as the trabecular thickness (Table 1). Although there were no significant differences among the four calcium‐fortified groups, rats fed Y‐Ca and Y‐I‐Ca had a trend towards higher levels of trabecular bone volume, higher ratio of trabecular bone volume/tissue volume, larger trabecular thickness, and lower trabecular separation compared to the Ca‐N group and the M‐Ca group (Table 1) that in turn showed the same trends as compared to the Ca‐D group. Calcium supplementation also showed a trend toward increasing the cortical bone volume and ratio of cortical bone volume/tissue volume with respect to the Ca‐D group. Moreover, the cortical thickness tended to be larger for rats fed M‐Ca, Y‐Ca, and Y‐I‐Ca as compared to both Ca‐D and Ca‐N.

### 2.2 Gut Metabolome

**Figure 1:**
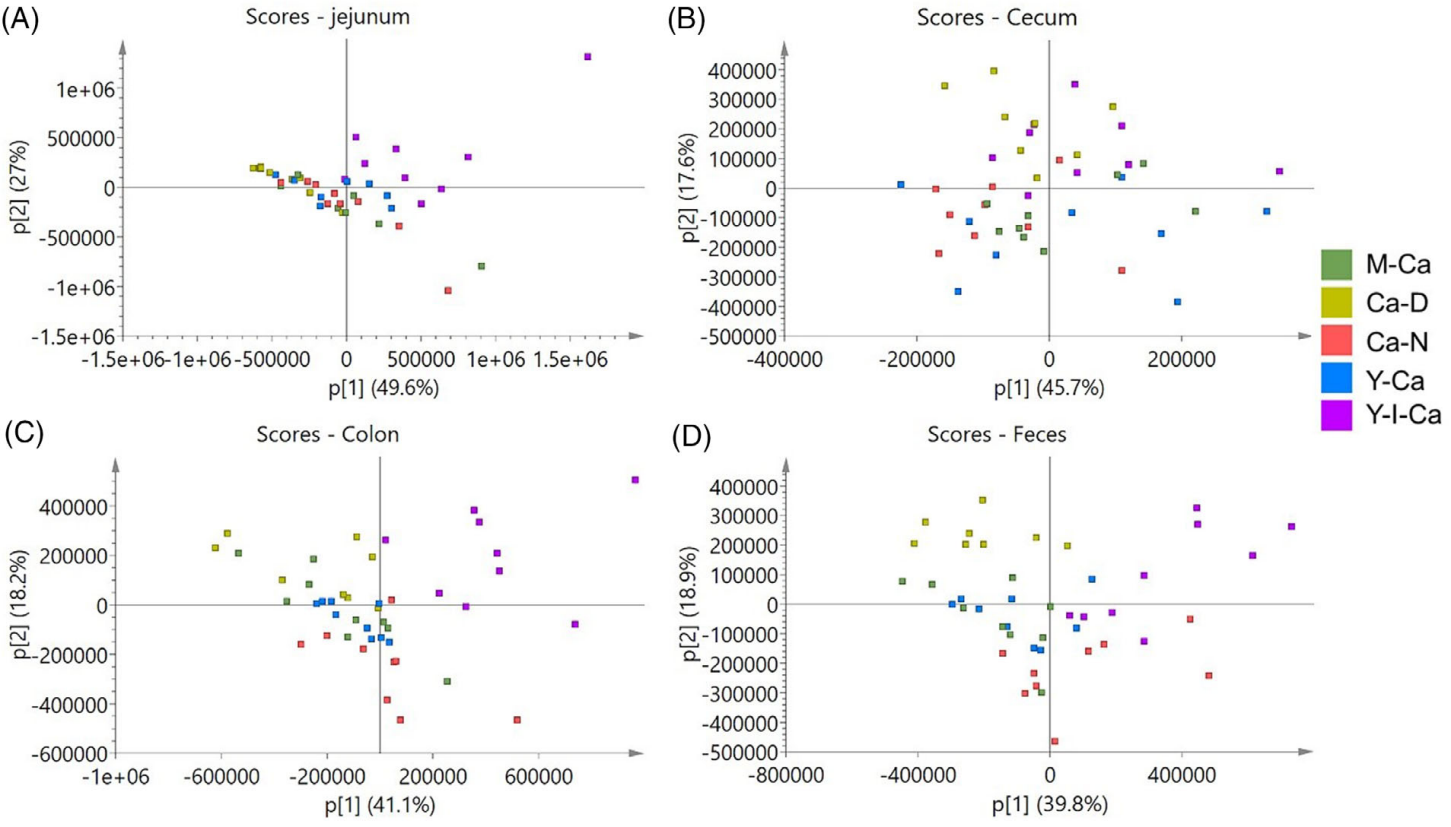
Score plots of PCA for all NMR metabolite profiles (*n* = 44) from jejunal content A), cecal content B), colon content C), and feces D). All score plots show principal component (PC) 1 versus PC 2 and axes indicate the percentage of total variation explained by each of the first two scores.

PCA was conducted to obtain an overview of all the metabolomes derived from the NMR spectra. Overall, rats fed a calcium‐deficient diet (Ca‐D) or inulin‐fortified diet (Y‐I‐Ca) were discriminated from rats fed other diets in all intestinal segments and feces (**Figure** **1**). OPLS‐DA models were performed to discriminate the NMR metabolite profiles between groups (Table S2, Supporting Information). In the jejunal content, the NMR metabolite profiles of Ca‐D versus Ca‐N, Y‐Ca versus Ca‐N, and Y‐Ca versus Y‐I‐Ca were discriminated by OPLS‐DA (*Q* ^2^ > 0.5) (Table S2, Supporting Information). In the cecal content and feces, OPLS‐DA models discriminated some paired groups (*Q* ^2^ > 0.5), including Ca‐D versus Ca‐N, Y‐Ca versus Ca‐N, M‐Ca versus Ca‐N, and Y‐Ca versus Y‐I‐Ca (Table S2, Supporting Information). Specific differences between these paired groups are shown in corresponding S‐line plots (Figures S3–S6, Supporting Information). The OPLS‐DA models failed to discriminate the NMR metabolite profiles of the M‐Ca group and the Y‐Ca group in any of the intestinal segments nor in feces.

**Figure 2:**
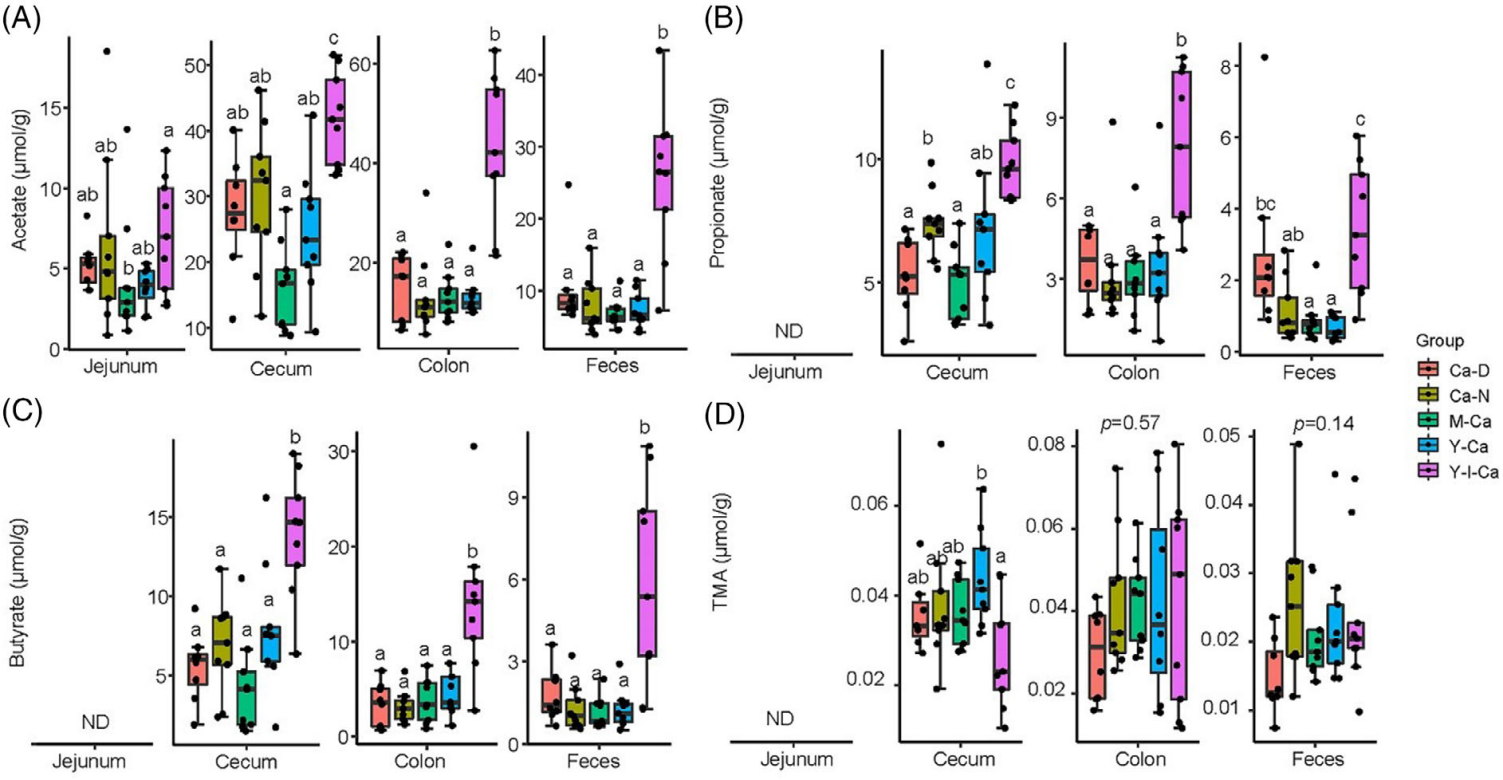
Concentrations (mean ± SD) of gut metabolites. The concentrations of acetate A), propionate B), butyrate C), and trimethylamine (TMA) D) in jejunal content, cecal content, colon content, and feces. ND, not determined. Different letters indicate significant differences (*p* < 0.05).

The concentrations of SCFAs (acetate, propionate, and butyrate) were quantified from the NMR data (**Figure** **2**A–C). Inulin fortification significantly increased acetate concentration in jejunal content and all three SCFAs (acetate, propionate, and butyrate) in distal gastrointestinal tract and feces. Accompanying this, the lowest pH in intestinal contents and feces was observed in the Y‐I‐Ca group (Figure S7, Supporting Information). In addition, the Ca‐N and M‐Ca groups and in particular the Y‐Ca group showed lower pH in the jejunal and cecal contents in comparison to the Ca‐D group (Figure S7, Supporting Information). Pearson correlation analysis showed that cecal butyrate/propionate was positively correlated with total BMD and/or spine BMD (Figure S8, Supporting Information).

Trimethylamine (TMA) was also quantified because it has been reported to be associated with bone metabolism in former studies^[^ ^19^, ^20^ ^]^ (Figure 2D). Rats fed Y‐I‐Ca had a lower concentration of TMA compared to the Y‐Ca group (*p <* 0.05) in cecal content, while there were no significant differences between groups in other intestinal segments and feces (Figure 2E).

### 2.3 Serum Metabolome

**Figure 3:**
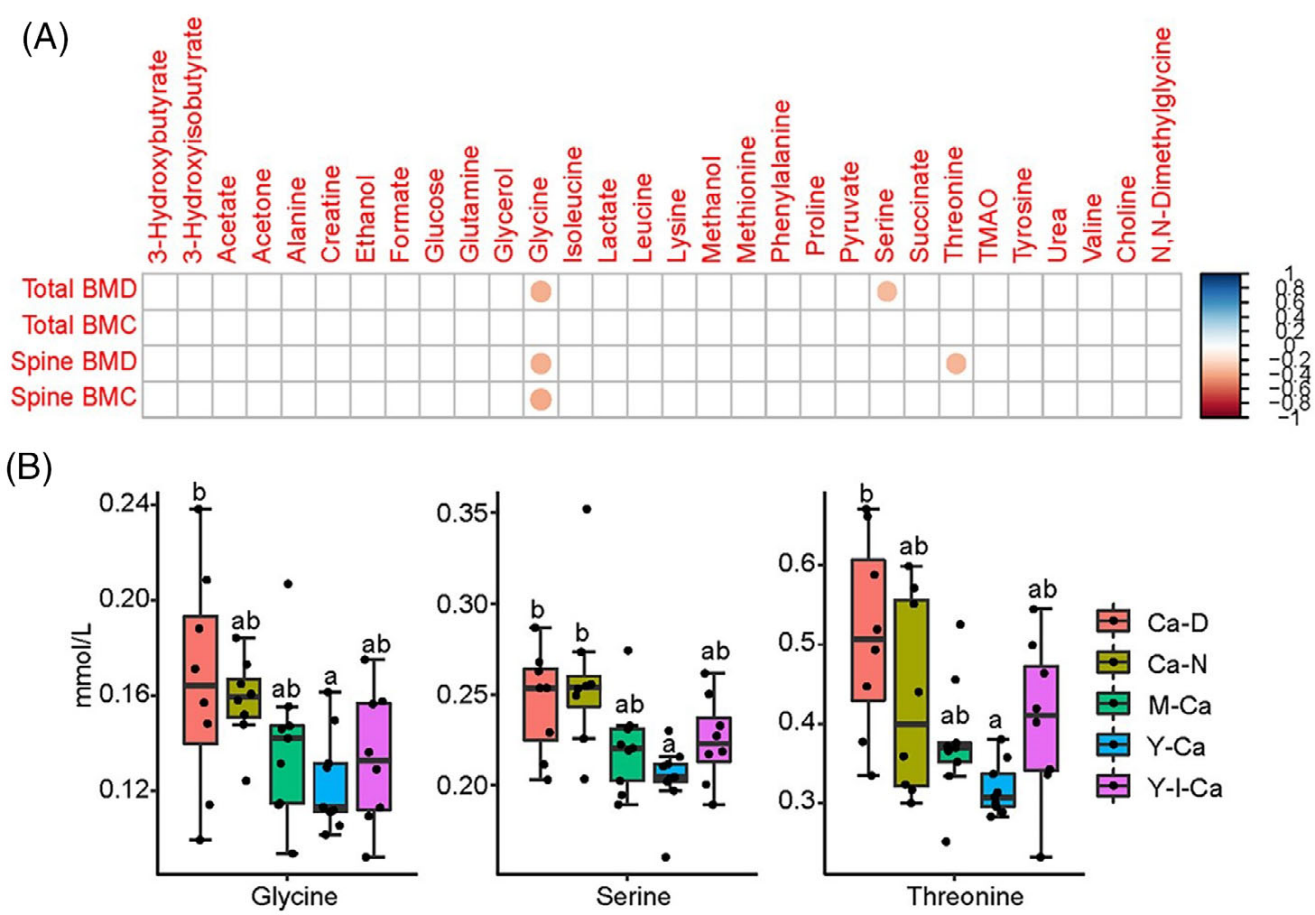
Serum metabolites. A) The Pearson correlations between NMR‐derived serum metabolites and bone parameters. Significant correlations (*p* < 0.05) are marked with colored circles, while insignificant correlations are left blank. B) The concentrations (mean ± SD) of serum glycine, serine, and threonine.

The concentrations of serum metabolites were quantified from the NMR data. Pearson correlation analysis between serum metabolites and bone parameters showed that the concentrations of three amino acids (glycine, serine, and threonine) were negatively correlated with spine BMD and/or total BMD (**Figure** **3**A). Intriguingly, rats fed Y‐Ca had lower concentrations of serum glycine, serine, and threonine compared to the Ca‐D and/or the Ca‐N group (Figure 3B).

Serum trimethylamine N‐oxide (TMAO) was also quantified as it has been associated with bone mineralization.^[^ ^20^ ^]^ However, no significant effect of diet on serum TMAO was found (Figure S9, Supporting Information).

PCA (Figure S10, Supporting Information) and OPLS‐DA (*Q* ^2^ < 0.5) of the complete serum NMR metabolite profiles could not discriminate between different diet groups.

### 2.4 Gut Microbiome Composition

**Figure 4:**
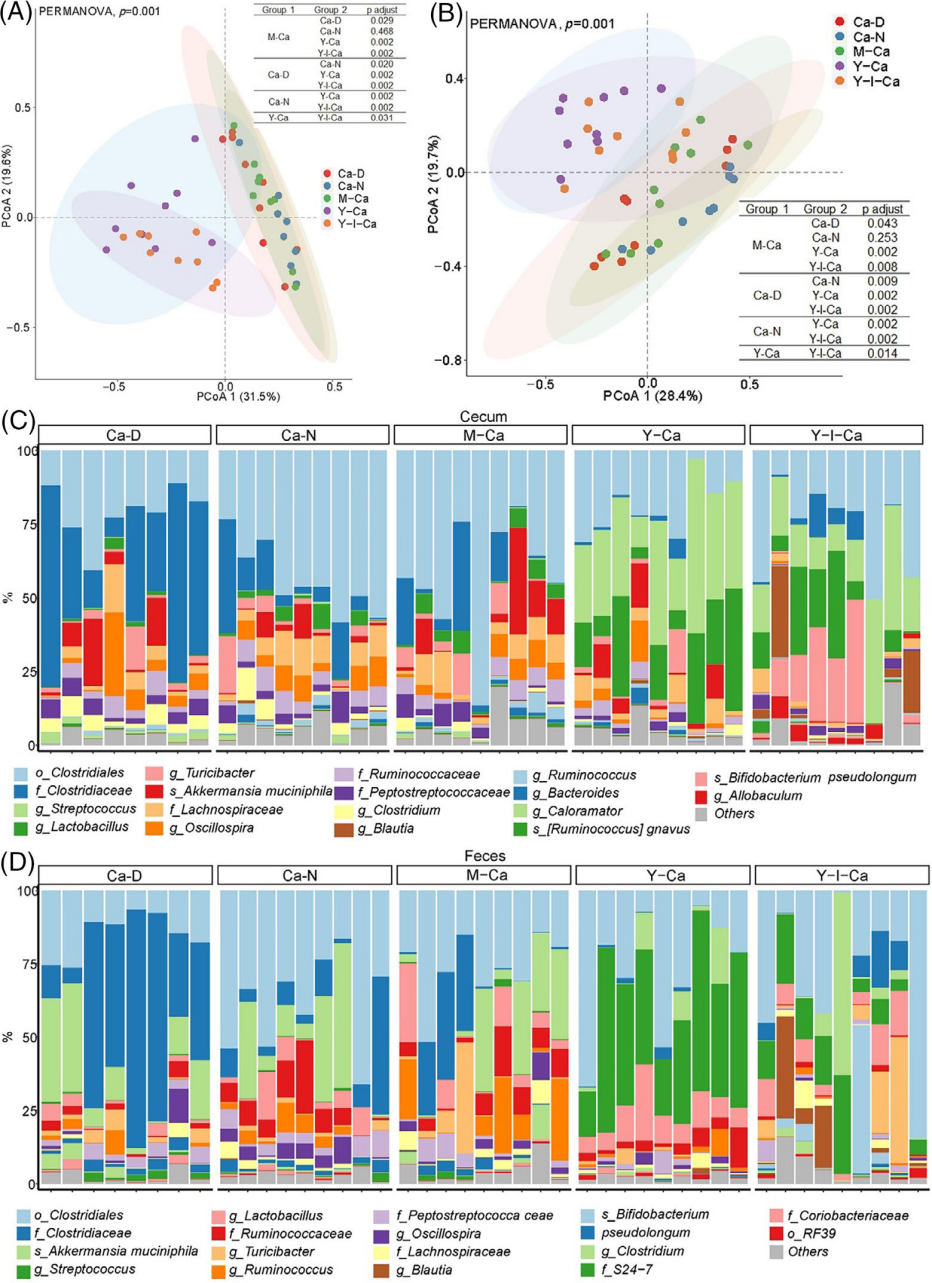
Principal coordinate analysis (PCoA) matrices of beta diversity of bacteria based on 16S rRNA sequencing in A) cecal content and B) feces. Inserted tables show the dissimilarity between paired groups in beta bacterial diversity assessed by PERMANOVA. The top 18 bacteria at the species level in C) cecal content and D) feces.

For the alpha diversity (to assess microbiome diversity within a sample), rats fed Y‐I‐Ca had the lowest observed index compared with the other four diets (*p* < 0.05) (Figure S11, Supporting Information). For the beta diversity (to assess between sample diversity; i.e., to assess the overall structure of the microbial community), the PCoA and PERMANOVA analysis suggested that there were significant differences in gut microbiome composition between groups (except the M‐Ca group vs the Ca‐N group) in both cecal content and feces (**Figure** **4**A,B).

For gut microbiome composition, collapsed features in different rat groups were summarized at the species level (Figure 4C,D). Contrary to yogurt‐free groups (the Ca‐D group, the Ca‐N group, and the M‐Ca group), *Streptococcus* predominated in rats fed Y‐Ca or Y‐I‐Ca (Figure 4C,D) which also had a higher relative abundance of *Lactobacillus* (Figure 4C). Rats fed Y‐I‐Ca had the highest relative abundances of *Bifidobacterium pseudolongum*, *Turicibacter*, *Blautia*, and *Allobaculum* in cecal content and/or feces (Figures 4C,D and S12, Supporting Information). Compared with the Ca‐D group, the Ca‐N group had a higher relative abundance of *Clostridiales* and a lower relative abundance of the *Clostridiaceae* in both cecal content and feces (Figure 4C,D). In addition, there were significant differences in some non‐dominant bacteria between groups. Rats fed Y‐Ca showed a higher relative abundance of *Staphylococcus*, and the lower relative abundances of *02d06*, *Fusibacter*, *Clostridiaceae*, *Caloramator*, and *Clostridium* in cecal content/feces compared with other groups without yogurt supplementation (the Ca‐D group, the Ca‐N group, and the M‐Ca group) (Figures S12,S13, Supporting Information). A comparison of the Y‐I‐Ca group and the Y‐Ca group showed that inulin intake also significantly increased the relative abundances of *Atopobium* and *Veillonellaceae*, and reduced the relative abundances of *Anaeroplasma*, *RF39*, and *Staphylococcus* in cecal content/feces (Figures S12,S13, Supporting Information). Intriguingly, compared to the Ca‐D group, the Ca‐N group had significantly lower relative abundances of *Erysipelotrichaceae*, *Mucispirillum schaedleri*, *Bacteroides*, *Anaeroplasma*, *RF39*, *Enterococcus*, *Klebsiella*, and *SMB53*, and higher relative abundances of *02d06* and *Brevibacterium*, in cecal content/feces (Figures S12,S13, Supporting Information).

### 2.5 Associations between Gut Microbiome Composition and Spine BMD

**Figure 5:**
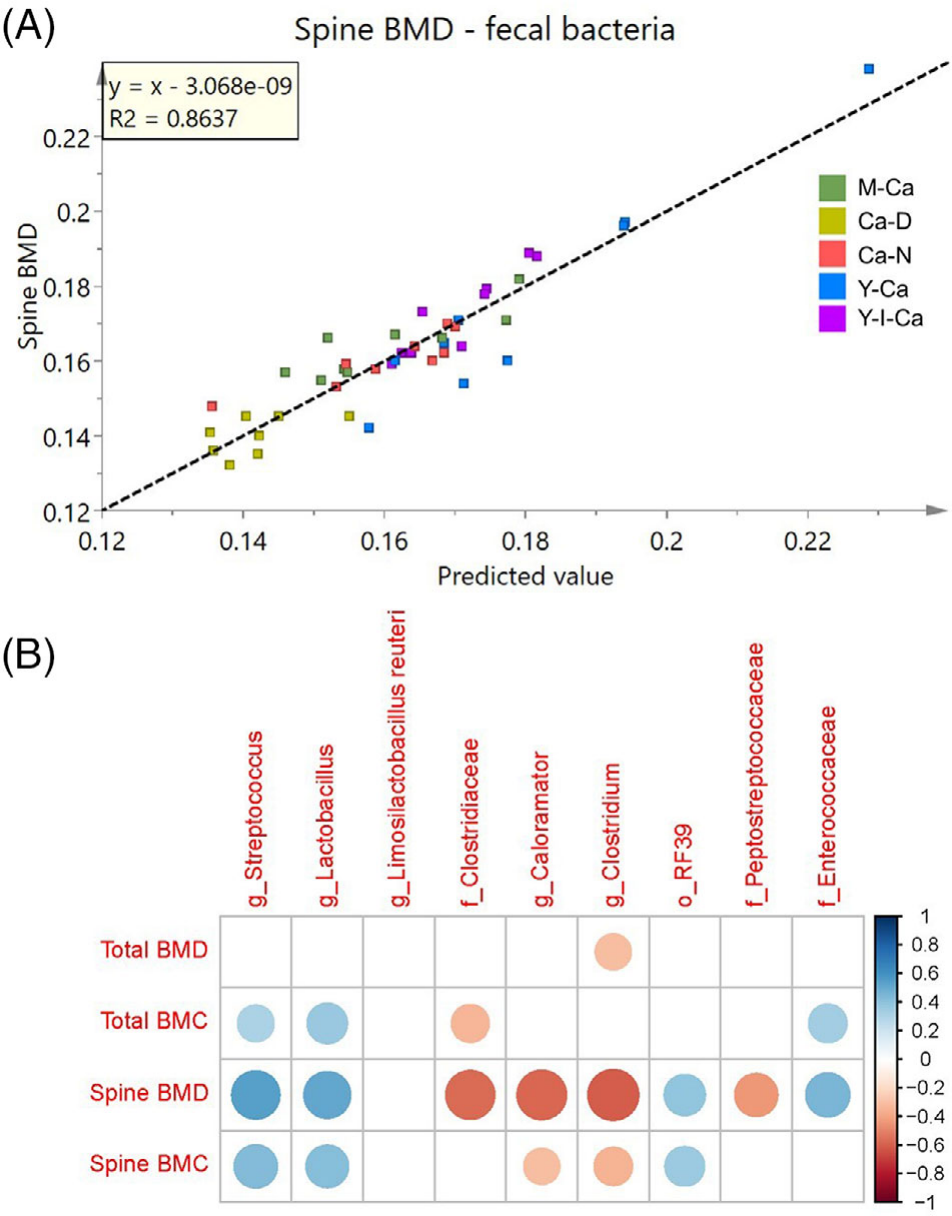
Associations between gut microbiota and spine BMD. A) The observed versus predicted plot of PLS model to predict spine BMD from fecal bacterial composition (*n* = 44) (*R* ^2^ = 0.86, *Q* ^2^ = 0.54). B) The Spearman correlations between bone parameters and several important bacteria. Significant correlations (*p* < 0.05) are marked with colored circles, while insignificant correlations are blank. The circle size and shade of color are proportional to the correlation coefficient.

In order to investigate a potential link between gut microbiome composition and bone mineralization, a PLS model was constructed between fecal bacterial composition and spine BMD (*R* ^2^ = 0.86, *Q* ^2^ = 0.54), as shown in **Figure** **5**A. The corresponding VIP plot revealed some potential fecal bacteria that correlated with spine BMD (Figure S14A, Supporting Information). Furthermore, Spearman rank correlation analysis showed that spine BMD was positively correlated with *Lactobacillus* (Rho = 0.51), *Streptococcus* (Rho = 0.55), *RF39* order (Rho = 0.40), and *Enterococcaceae* (Rho = 0.46), and negatively correlated with *Clostridium* (Rho = −0.61), *Clostridiaceae* family (Rho = −0.56), *Caloramator* (Rho = −0.58), and *Peptostreptococcaceae* family (Rho = −0.43) (Figure 5B).

## 3 Discussion

### 3.1 Milk Calcium‐Fortified Yogurt Enhances Bone Mineralization Mediated through Stimulation of Beneficial Gut Bacteria

In this study, OVX rats fed Y‐Ca and Y‐I‐Ca showed higher spine BMD in comparison to the Ca‐N group and the M‐Ca group, suggesting that ingestion of yogurt has the potential to prevent bone demineralization in postmenopausal women as also found in clinical studies.^[^ ^11^, ^21^ ^]^ Addition of inulin did not augment this beneficial function of calcium‐fortified yogurt. While we observed a significant difference between the Y‐Ca group and the Ca‐N group in spine BMD and BMC, this was not reflected in the total BMD and BMC, mechanical femur strength, or femoral micro‐structure. X‐ray µCT revealed that milk calcium‐fortified yogurt supplementation showed a trend to increase trabecular bone volume of the femoral metaphysis, but not the cortical bone volume, indicating that the higher proportion of trabecular bone (≈80%) in the spine compared to other bone sites (e.g., ≈20% in the femur) may contribute to a more rapid response during an intervention.^[^ ^1^, ^14^ ^]^

In our study, ingestion of calcium‐fortified yogurt significantly modulated the gut microbiome. A PLS model revealed a strong correlation between the fecal microbiome and spine BMD, and correlation analysis revealed that this could be ascribed to abundances of *Lactobacillus* and *Streptococcus* (Figure 5B). These findings support previous work showing that lactobacilli (e.g., *L. reuteri* ^[^ ^22^ ^]^ and *L. rhamnosus* ^[^ ^6^ ^]^) treatment could suppress bone loss in estrogen‐deficient mice. Studies indicated that the beneficial effects of *Lactobacillus* supplementation on bone mass may be associated with increased generation of SCFAs in the gut.^[^ ^23^, ^24^ ^]^ However, the results here do not support this mechanism, indicating that other potential mechanisms related to *Lactobacillus* may play a crucial role in promoting bone mineralization in OVX rats. Li et al.^[^ ^6^ ^]^ found that lactobacilli could attenuate bone loss in OVX mice by preventing estrogen deficient‐induced increase in intestinal permeability.

### 3.2 Gut–Bone Associations May also Involve Suppression of Harmful Gut Bacteria

Both the use of antibiotics and germ‐free conditions have been found to increase BMD of mice,^[^ ^6^, ^25^ ^]^ indicating that blocking the activities of some bacteria might be an effective target for gut‐modulating foods to improve bone health. In the present study, the decreased *Clostridiaceae* family and its two specific genus (*Clostridium* and *Caloramator*) in the Y‐Ca group was negatively correlated with spine BMD, indicating that the beneficial effect of yogurt on spine BMD might be associated with inhibition of some bacteria from the *Clostridiaceae* family. A higher abundance of *Clostridium* has been observed in rats OVX rats after ovariectomy^[^ ^26^ ^]^ and in postmenopausal women with osteoporosis as compared to osteopenia (pre‐symptoms of osteoporosis),^[^ ^27^ ^]^ indicating that *Clostridium* may be associated with bone loss and osteoporosis risk in postmenopausal women. Hou et al.^[^ ^28^ ^]^ also observed a negative correlation between the relative abundance of the *Clostridiaceae* family and BMD in humans. However, limited evidence currently exists to confirm that the reduced levels or *Clostridium*, *Caloramator*, and the *Clostridiaceae* family can prevent estrogen deficiency‐induced bone loss.

### 3.3 Glycine, Serine, and Threonine Metabolism as a Potential Mechanism Affecting Bone Mineralization

In this study, lower concentrations of especially serum glycine, but also serum serine and threonine in the Y‐Ca group were associated with an increased spine/total BMD (Figure 3A), suggesting that the beneficial effect of milk calcium‐fortified yogurt on bone mineralization may involve glycine, serine, and threonine metabolism. Zhang et al.^[^ ^29^ ^]^ recently reported that the glycine, serine, and threonine metabolism was associated with BMD in a clinical cohort study, with a negative correlation between glycine and the lumbar spine BMD. In addition, Xu et al.^[^ ^30^ ^]^ found that a beneficial effect of lactoferrin on bone formation of rats also involved the glycine, serine, and threonine metabolism. A possible link between the glycine, serine, and threonine metabolism and bone metabolism involves collagen, the most abundant protein in the bone matrix, consisting of repeating units of Gly‐Pro‐X. A cross‐sectional study in discordant monozygotic twins showed that an increased intake of glycine resulted in increased BMD.^[^ ^31^ ^]^ However, how yogurt ingestion in the present study affected the glycine, serine, and threonine metabolism, apparently related to collagen metabolism in bone, remains unknown.

### 3.4 Inulin Shapes the Gut Microbiome but without Apparent Effects on Bone Mineralization

Inulin significantly changed the gut metabolome and modulated the gut microbiome composition (e.g., increased relative abundance of *B. pseudolongum*, *Turicibacter*, *Blautia*, and *Allobaculum*) in the present study, consistent with observations in previous studies.^[^ ^32^, ^33^ ^]^ However, changes in the gut environment caused by the combination of inulin and milk calcium‐fortified yogurt had no additive effect on BMD compared to only milk calcium‐fortified yogurt supplementation in this study. Previous studies suggested that *Turicibacter*, *Blautia*, and *Allobaculum* were positively correlated with BMD in certain specific situations,^[^ ^27^, ^34^, ^35^ ^]^ but these links have not been confirmed by direct studies.

Microbial metabolites are potential mediators affecting bone metabolism and bone health.^[^ ^9^ ^]^ Inulin‐derived SCFAs, especially butyrate, may be a crucial bacterial‐derived metabolite that can contribute to an improved bone health.^[^ ^23^, ^36^, ^37^ ^]^ SCFAs can act as substrates for the colonic epithelium,^[^ ^38^ ^]^ stimulating its growth and/or activity, thereby enhancing calcium absorption in the distal GI tract.^[^ ^39^ ^]^ Another underlying mechanism may involve that gut‐derived butyrate reaches the distal bone and directly participate in the regulation of bone metabolism‐associated Terg differentiation.^[^ ^23^ ^]^ However, in the present study the higher level of butyrate in the Y‐I‐Ca group did not increase bone mineralization as compared to the Y‐Ca group. A possible explanation is that the distal GI tract is responsible for less than 10% of calcium absorption,^[^ ^40^ ^]^ and calcium absorption may be highly efficient in rat fed the Y‐Ca diet. Moreover, even though increased in the content of the GI tract, the level of circulating butyrate in the Y‐I‐Ca may be too low to exert a regulatory function on bone homeostasis. Recent in vitro studies suggested that butyrate supplementation could directly stimulate bone metabolism‐associated Treg differentiation, when using a physiological unrealistically high concentration of butyrate (e.g., 100 µM).^[^ ^36^, ^41^ ^]^ Positive effects of gut‐derived SCFAs on bone mineralization may also be related with hormonal effects.^[^ ^42^ ^]^ Li et al.^[^ ^36^ ^]^ reported that nutritional supplement of butyrate synergistically increased the beneficial effect of intermittent parathyroid hormone (iPTH) on bone formation, but did not directly improve bone health of germ‐free mice without the treatment of iPTH.

### 3.5 Differences between Milk and Yogurt in Bone Health

Calcium supplementation showed a pronounced effect on BMD and BMC, but while milk calcium‐fortified yogurt intake did promote bone mineralization, milk calcium‐fortified milk did not. In addition to modulating gut microbiome, our data also revealed that yogurt consumption was associated with a pH‐lowering effect in the proximal part of the intestine (Figure S7, Supporting Information). This finding is noteworthy as it has been reported that solubility of calcium phosphate (dairy calcium) is highly pH‐dependent.^[^ ^43^ ^]^ Thus, it is plausible that the beneficial effect of yogurt on bone mineralization as compared with milk also can be ascribed to a higher bioavailability of calcium in the proximal part of the intestine where the majority of calcium absorption is taking place. A recent meta‐analysis found that a definitive conclusion on a beneficial effect of milk on bone health could not be drawn when diets were adjusted for calcium intake.^[^ ^44^ ^]^ Therefore, a positive correlation between milk intake and BMD in many epidemiological observational studies is likely associated with the rich calcium content in milk rather than milk as delivery matrix.^[^ ^45^ ^]^ A cross‐sectional study based on 4310 Irish older adults (>60 years) showed that yogurt consumption was positively correlated with BMD in three different regions (hip, femoral neck, and vertebra) in women but not in men, while milk consumption was not significantly associated with changes in BMD and bone turnover markers.^[^ ^46^ ^]^ Our results provide further evidence to support that yogurt may be a more effective calcium vehicle than milk to attenuate bone loss in postmenopausal women.

### 3.6 Study Limitations

In this study, a dairy calcium ingredient was added into milk and yogurt to obtain a calcium content of the diets meeting the recommended dose for rats. The same level of fortification was used for the milk and yogurt diets, and the two diets are therefore comparable in means of origin and quantity of the included dairy calcium. Even though the fortified calcium source was of dairy origin, whether a fortification has an effect on the general bioavailability of calcium as compared to milk and yogurt that have not been fortified, remains unknown. For practical reasons diets were served to the rats directly from frozen storage to limit bacterial growth and replaced after 24 h. We carefully inspected the diets during serving to the rats to ensure that the diets did not undergo spoilage during the 24 h (Figure S15, Supporting Information). However, as we did not make microbial analyses of the products after 24 h at room temperature, we cannot exclude a potential difference in bacterial growth between milk‐ and yogurt‐containing diets. Unfortunately, it was not possible to quantitatively record left‐over foods and exact feed intake therefore remains unknown. However, our qualitative assessment indicated that there was no systematic association between type of diet (treatment group) and amount of diet left over.

## 4 Conclusion

Our findings suggest that the beneficial effects of yogurt consumption on spine BMD are likely associated with a proliferation of lactobacilli and potentially also an inhibition of *Clostridiaceae* members and a lowering effect of pH in the proximal part of the intestine. Concomitantly, the present study revealed a link between enhanced spine BMD and the glycine, serine, and threonine metabolism, although the potential mechanism remains unknown. Gaining a further understanding of whether an effect on glycine‐related pathways can be considered a marker of bone mineralization or whether a direct link exists between gut microbial activity and glycine metabolism could expand future opportunities for development of strategies in prevention of bone demineralization.

## 5 Experimental Section

### Experimental Diets

Diets were prepared in the application center of Arla Food Ingredients (Viby, Denmark). The food paste for the five different groups were produced by mixing the dry ingredients (Altromin powdered diet and dairy calcium (Capolac MM‐0525 BG)) with the corresponding wet component (water, milk, or yoghurt), as shown in Table S1, Supporting Information. Detailed procedures for diet preparation were provided in the supplementary file (Method S1, Supporting Information). Calcium content and milk/yogurt/inulin addition were the main differences in composition among the five paste diets: 1) Calcium‐deficient diet (Ca‐D): 0.04% w/w calcium; 2) Normal calcium diet (Ca‐N): 0.26% w/w calcium; 3) Milk calcium‐fortified milk diet (M‐Ca): 0.28% w/w calcium, 49.6% w/w skimmed milk; 4) Milk calcium‐fortified yogurt diet (Y‐Ca): 0.28% w/w calcium, 49.6% w/w yogurt (fermented skimmed milk); 5) Milk calcium‐ and inulin‐fortified yogurt diet (Y‐I‐Ca): 0.28% w/w calcium, 49.6% yogurt, and 2.5% w/w inulin.

### Animals

Fifty‐five 6‐week‐old female NTac:SD (Sprague‐Dawley (SD)) rats (Taconic Biosciences, Ll. Skensved, Denmark) were purchased and housed in 1354 G cages with high lids (Tecniplast, Buguggiate, Italy) and aspen bedding (Tapvei, Paekna, Estonia) with three or four rats per cage. Cages were enriched with a cardboard igloo, aspen biting sticks, and an aspen tunnel (Brogaarden, Lynge, Denmark). Health monitoring was performed according to FELASA guidelines.^[^ ^47^ ^]^ All rats were allowed ad libitum access to a standard diet (Altromin 1324, Brogaarden) for 1 week to adapt to the new housing environment before aseptically ovariectomy was performed through the flank under anesthetization with 0.2 mL g^−1^ body weight (BW) with 25% Midazolam (5 mg mL^−1^ midazolam, B. Braun, Melsungen, Germany) and 25% Hypnorm (0.315 mg mL^−1^ of fentanyl citrate and 10 mg mL^−1^ of fluanisone, Skanderborg Pharmacy, Denmark). Subsequently, 44 recovered OVX rats were randomly allocated to 15 cages, three rats per cage (except for one cage that only accommodated two rats), and allowed ad libitum access to one of five experimental diets (three cages per group) for 6 weeks. The experimental diets were refreshed every 24 h, and BW of the rats was recorded once a week. After the 6‐week dietary intervention, all rats were anesthetized, and heart blood samples were collected before head decapitation. Finally, jejunal content (10 cm up the cecum), cecal content, colon content (8 cm down the cecum), and feces of all rats were collected and stored at −80 °C for 16S RNA sequencing or −20 °C for metabolome analysis. The rat bodies were stored at −20 °C for 1 month until the measurement of bone parameters.

The rat study was authorized by the Animal Experiments Inspectorate in Denmark (License No. 2020‐15‐0201‐00434) and implemented at Department of Veterinary and Animal Sciences Copenhagen University in accordance with the Directive 2010/63/EU on protection of animals used for scientific purposes.

### Bone Parameters

DXA scanning was performed to measure the bone mineral density (BMD) and bone mineral content (BMC) of rats by scanning the whole body using a 1.8 μGy scanner (1.8 μGy, Lunar Prodigy, GE Health Care, Chicago, IL, USA). Total BMD and BMC as well as spine BMD and MBC were determined,^[^ ^14^, ^48^ ^]^ to evaluate bone mineralization.

Three‐point bending test was employed to evaluate the mechanical strength of femur bone in a TMC‐touch texture analyzer (Food Technology Corporation, VA, USA). The space between the two supporting points was 20 mm, and the femur was placed at the two points. Subsequently, the downward mid‐diaphysis with a speed of 1.0 mm min^−1^ was loaded at the midpoint of the femur to measure the power at fracture.

X‐ray micro‐computed tomography (µCT) was measured in an Xradia 620 Versa X‐ray microscope (Zeiss, Germany) to evaluate potential changes in the microstructure of the distal femoral metaphysis. The detailed method was provided in the supplementary file (Method S2, Supporting Information).

### Proton (^1^H) NMR Spectroscopy

Sample preparation for pH measurement and NMR spectroscopy was performed according to previously published methods.^[^ ^49^ ^]^ The detailed methods for sample preparation (intestinal contents, feces, and serum) were provided in supplementary material (Method S3, Supporting Information). ^1^H NMR spectra were acquired using a Bruker Avance IVDr NMR spectrometer equipped with a 5 mm ^1^H‐optimized double resonance broad‐band probe operating at a frequency of 600.13 MHz (Bruker BioSpin, Rheinstetten, Germany). A one‐dimensional NOESY pulse sequence was adopted to acquire the ^1^H NMR spectra with specific parameters including measurement temperature: 300 K, relaxation delay: 4 s, spectral width: 7212 Hz, data points: 32k, and scans: 32. A line‐broadening factor of 0.3 Hz was employed to process free induction decays before Fourier transformation. All spectra were baseline and phase corrected before further analysis. Identification and quantification of metabolites from the obtained NMR spectra were implemented in Chenomx (Version 8.6, Chenomx Inc., Alberta, Canada).

### DNA Extraction, Amplicon Sequencing, and Bioinformatics

Gut microbiome composition was analyzed according to the method described by He et al.^[^ ^49^ ^]^ with slight modifications. Briefly, DNA was extracted from 100 mg cecal content or feces using the Micro Bead beat AX kit (A&A Biotechnology, Poland). Amplicon sequencing was prepared from 1 ng µL^−1^ DNA with a two‐step PCR method targeting the 16S rRNA gene V3 hypervariable region (forward primer NXt_338_F and reverse primer NXt_518_R). The PCR products were purified with Agencourt AMPure XP Beads (Beckman Coulter Genomics, 245, MA, USA) and pooled in equimolar for NextSeq‐based (Illumina, CA, USA) high throughput sequencing. For bioinformatics, raw datasets were merged and trimmed using ‐fastq_minovlen 100, ‐fastq_maxee 2.0, ‐fastq_truncal 4, ‐fastq_minlen 130. The UPARSE pipeline was employed to purge the dataset and construct de novo Operational Taxonomic Units (OTU), and then SINTAX was used to predict taxonomy. Alpha and beta diversity analyses were based on a contingency table rarefied to 23 000 (feces) and 14 000 (cecum content) reads per sample, and pairwise wilcoxon test and PERMANOVA were performed to determine statistically significant differences in diversity metrics. Responding taxonomic features were extracted through two‐group comparison using DESeq2^[^ ^50^ ^]^ with a preset adjusted *p*‐value of 0.05. Data visualization were mainly performed in R3.6.2 (RStudio, MA, USA) by using a “ggplot2” package.^[^ ^51^ ^]^

### Multivariate Data Analysis (MVDA)

To reveal the differences in the gut metabolome or gut microbiome between dietary groups, multivariate data analysis (MVDA) was performed in SIMCA 16 (Sartorius, Umeå, Sweden) or R by using a package of “vegan.”^[^ ^52^ ^]^

Prior to MVDA, baseline and phase‐corrected NMR spectra were processed with the following steps: alignment, removal of the outer spectral regions (<0.6 and >9.0 ppm) and the region containing the residual water signal (4.76–4.90 ppm), normalization (to sample weight), and binning using a bin width of 0.005 ppm. Pareto scaling was conducted to scale these binning variables before MVDA. Principal component analysis (PCA) was performed to obtain an overview of all NMR profiles. Furthermore, to revel differences between two treatment groups, orthogonal projections to latent structures discriminant analysis (OPLS‐DA) was employed. The S‐line plot of OPLS‐DA was used to elucidate specific differences between two groups. Partial least squares regression (PLS) was employed to build the relation between two sets of variables. Variable importance for the projection (VIP) and the coefficients between specific variables were used to illustrate the important variables to explain *X*‐variables and to correlate *Y*. The predictability (*Q* ^2^) of PCA, OPLS‐DA, and PLS model were evaluated by 7‐fold cross‐validation that leaves out one observation and fits the model with the remaining six observations every round (seven rounds in total). To avoid overfitting, permutations plot was employed to assess the model rationality. Principle coordinate analysis (PCoA) was used to cluster the β‐diversity of bacteria based on the dissimilarity matrix generated by weighted‐UniFrac method.

### Statistical Analysis

All results were reported as mean ± standard deviation (SD). One‐way ANOVA followed by Tukey's post hoc was employed to compare the mean values between groups in Origin 2018 (OriginLab Corporation, MA, USA). If not normally distributed and homogeneous in the variances, data were analyzed by the Kruskal–Wallis test followed by paired multiple comparisons with Bonferroni correction. Correlation analysis was conducted in R by using a package of “Hmisc.”^[^ ^53^ ^]^ For the correlations between gut microbiota and BMD/BMC, Spearman rank correlation was conducted. Pearson correlation analyses were conducted to examine the correlations between bone parameters and other parameters. If variables were not normally distributed (Shapiro‐Wilk test), variables were log‐transformed before Pearson correlation analysis. For bacterial data and correlation analysis, the false discovery rate (FDR) method was conducted to adjust *p* values. A *p* value of 0.05 was considered the significance threshold.

## Conflict of Interest

A. Andersen is employed by Arla Food Ingredients, and H.J. Andersen is employed by Arla Food amba. A.K. Hansen has collaborated with food and pharmaceutical enterprises (listed on https://ivh.ku.dk/english/employees/?pure = en/persons/107126). All other authors declare no conflicts of interest.

## Author Contributions

H.C.B. formulated the idea, W.H., D.S.N., H.J.A., A.K.H., and H.C.B. designed the animal study, A.A. developed diets, L.S.Z. and A.K.H. performed OVX, L.S.Z. supervised the animal study, W.H., Z.X., N.K.W., R.T., L.S.Z., and H.C.B. performed the experiments and analyzed the results, W.H., Z.X., N.K.W., A.A., H.J.A., H.B., D.S.N., A.K.H., and H.C.B. discussed the results, W.H. wrote the manuscript draft and all authors commented on the manuscript.

## Supporting information



Supporting Information

## Data Availability

The data that support the findings of this study are available from the corresponding author upon reasonable request.
