# Supplementary material for: Yogurt Benefits Bone Mineralization in Ovariectomized Rats with Concomitant Modulation of the Gut Microbiome
Source: Mol Nutr Food Res. 2022 Sep 15;66(20):2200174. doi: 10.1002/mnfr.202200174 (PMC9788323; doi:10.1002/mnfr.202200174)
Supplement: Supplementary file 1 — Supporting Information [file MNFR-66-2200174-s001.pdf]

Supplementary files

Method S1. Preparation of food pastes

The yoghurt base was produced from fresh skimmed milk, which was homogenized (200/50 bar) and pasteurized (72 ºC, 15 s) prior to fermentation with culture YC-X11 (Chr. Hansen A/S, Denmark) at 42 ºC until pH 4.5 was reached. The product was cooled to 5 ºC and stored at 4 ºC until production of food paste the next day. The food pastes for the five different groups were produced by mixing the dry ingredients (Altromin powdered diet and Capolac® MM-0525 BG) with the corresponding wet component (water, milk or yoghurt), see Table S1.

The dry ingredients for the recipes containing milk mineral (Capolac®) were mixed using an Inversia Tumbler Mixer equipped with a 20 liter drum (25 rpm for 25 min) to ensure a homogeneous distribution of the component in the powder prior to mixing the food paste. The diets without milk mineral were prepared from dry ingredients as delivered by Altromin.

For each recipe, the wet component was added to a Stephan Cooker UM/SK 44 equipped with a sharp knife mixing system. The dry ingredient mix was added and the paste was mixed for 2 min at 100% speed and 3000 rpm. The product was checked and mixing was continued for 5 min at 50% speed.

After mixing, 150 ± 1 g paste was transferred to 140 mL aluminum beakers with snap-on PET lids and stored at -18 ºC until served. Microbiological evaluations of the frozen products were conducted prior to release from Arla Foods Ingredients.

Table S1. Ingredient composition of the five different food pastes (%)

Ingredients	Ca-D	Ca-N	M-Ca	Y-Ca	Y-I-Ca
Tap water	50	50			
Altromin C1031(0.06% Calcium)	50		49.646	49.646	
Altromin C1000 (0.5% Calcium)		50			
Skimmed milk*			49.646		
Capolac® (dairy calcium)			0.708	0.708	0.708
Yogurt				49.646	49.646
C1031: -5% saccharose +5% inulin					49.646
Nutritional compositions					
Fat (g/100g)	2.5	2.5	2.5	2.5	2.5
Protein (g/100g)	8.6	8.6	10.3	10.3	10.3
Carbohydrates (g/100g)	29.7	29.7	32.0	32.0	32.0
Water (g/100g)	54	54	50	50	50
Energy (Kcal/100g)	180.6	180.6	197.4	197.4	197.4
Calcium (g/100g)	0.04	0.26	0.28	0.28	0.28

* Skimmed milk: protein (3.5%), fat (0.03%), carbohydrate (4.7%), total solids (9.2%), calcium (0.124%), ash (0.7%), energy (33.07 kcal/100g)

Method S2. X-ray micro-computed tomography

The femora were immersed in 96% EtOH and wrapped in cotton wool to keep them stable during measurement. 7700-µm high volumes of interest (VOI) containing the distal metaphysis were measured. The instrument was operated at 60 kV and 6 W with the LE2 filter inserted for attenuation of low-energy X-rays. 1601 projections were recorded over a 360° sample rotation with 2 s exposure/projection and using the 0.4× objective and a detector binning of 2 to result in an isotropic voxel size of 7.6 µm. The reconstructed tomograms were processed with Dragonfly Version 2021.3 (Object Research Systems Inc, Montreal, Canada). 2200-µm high VOIs for analysis of trabecular bone were selected starting 1500 µm above the most distal part of the growth plate following the procedure described in reference[18]. Segmentation of trabecular bone was done by manually drawing along the endocortical surface of the cortex in every 30th image along the bone long axis followed by automatic interpolation and segmentation of marrow versus trabecular bone by Otsu's method. For analysis of cortical bone, VOIs starting from the most proximal part of the measured volume and extending 980 µm towards the distal end were segmented by Otsu's method to separate bone from marrow and surrounding EtOH. Trabecular bone was subtracted by the same semi-manual procedure described above. Trabecular and cortical bone micro-structural parameters were computed for each of the two segmented VOIs using Dragonfly's Bone Analysis Wizard.

Method S3. Preparation of intestinal contents, feces and serum for NMR spectroscop

Approx. 100 mg intestinal content or feces were mixed with 200 µL Milli-Q water in a 1.5 mL Eppendorf tube. The pH of the sample supernatant was measured by using a pH meter equipped with a semi-micro electrode (Radiometer PHM92, Copenhagen, Denmark) after vortexing for 15 sec and centrifugation at 4 °C at 10000 × g for 5 minutes. Subsequently, 600 µL phosphate buffer (0.75 mol/L, pH 7.4) in D_2O containing 3 mM NaN_3 was added to the sample to achieve a final ratio of intestinal sample weight to water volume of 1:8. The mixture sample was vortexed for 15 sec before centrifugation at 4 °C at 10000 × g for 5 minutes. To remove large molecule compounds, 500 µL supernatant was transferred to a 10 K Amicon Ultra (Merck Millipore Ltd., Cork, Ireland) and washed three times using ultrapure water and centrifugation at 4 °C at 14000 × g for 40 minutes. Finally, 400 µL filtrate, 170 µL D_2O, and 30 µL D_2O containing 0.05 wt% trimethylsilyl-propanoic acid (TSP) (Sigma-Aldrich) were added into a 5-mm NMR tube with a final TSP concentration of 0.160 mM.

For the preparation of serum samples, a volume of 400 µL serum was filtered with 10 K Amicon Ultra (Merck Millipore Ltd., Cork, Ireland) by centrifugation at 4 °C at 14000 × g for 120 min. Subsequently, 300 µL filtrate mixed with 200 µL phosphate buffer in D_2O (0.3 mol/L, pH 7.4, containing 3 mM NaN_3) and 25 µL D_2O containing 0.05 wt. % TSP were added into a 5-mm NMR tube.

Table S2. Q^2 of OPLS-DA models between groups for different intestinal contents and feces.

Comparison	Jejunum	Cecum	Colon	Feces
Ca-D VS Ca-N	0.87	0.83	0.83	0.90
Y-Ca VS Ca-N	0.68	0.72	0.24	0.76
M-Ca VS Ca-N	F	0.54	0.17	0.70
M-Ca VS Y-Ca	F	0.34	F	0.24
Y-I-Ca VS Y-Ca	0.75	0.86	0.56	0.81

F indicates that the number of components of the OPLS-DA model is 0, indicating that OPLS-DA could not discriminate the compared two groups.

Supplementary Figures

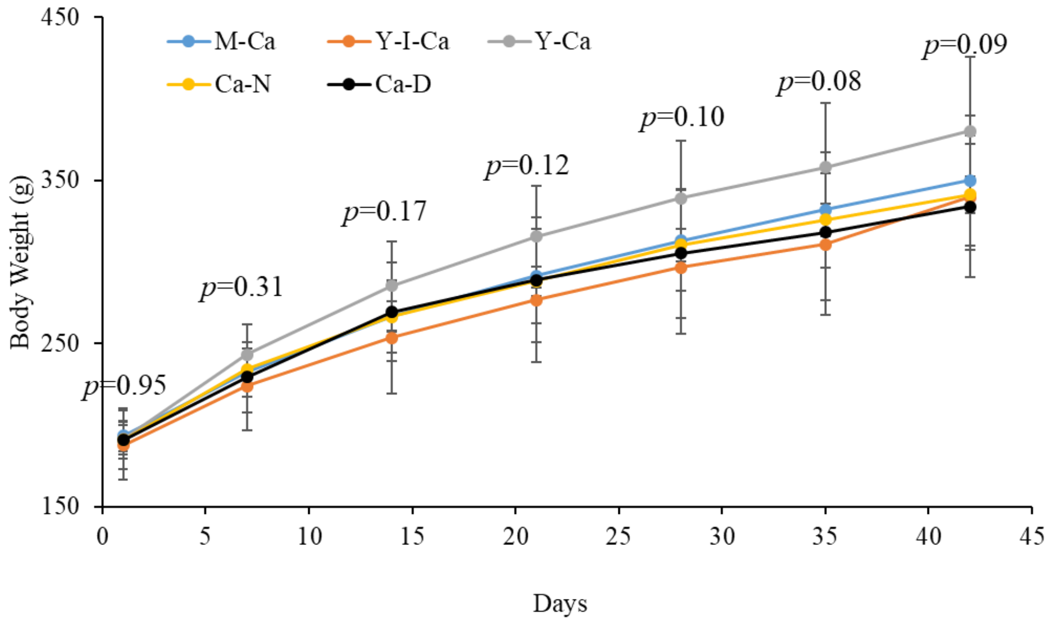

Figure S1. Body weight variations of OVX rats fed with five different diets during a 6-week intervention.

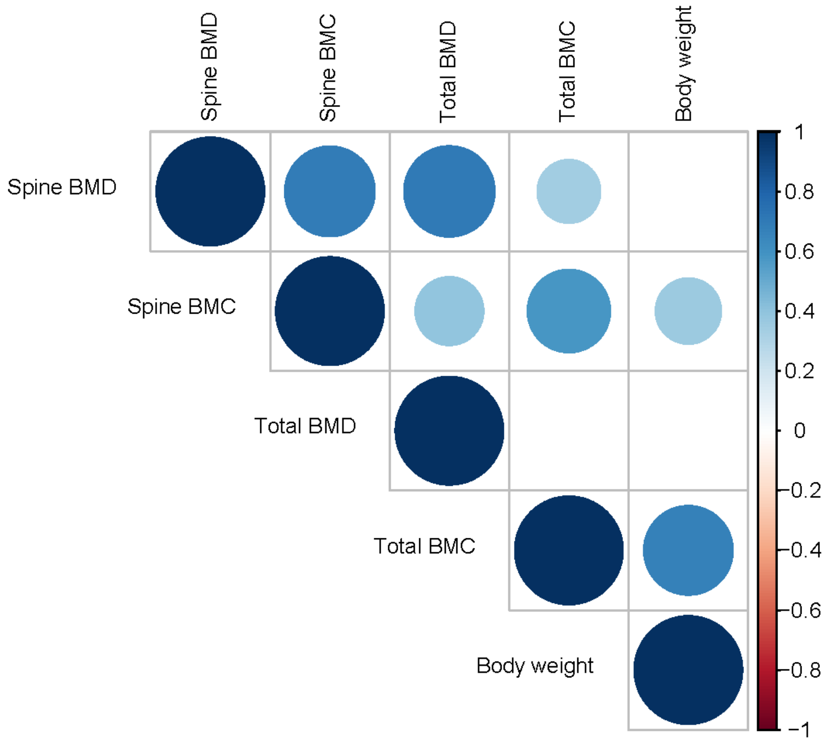

Figure S2. The correlations between bone parameters and body weight. The symbol circle represents significant correlations (p<0.05) between parameters, and the corresponding color shows the value of correlation coefficients.

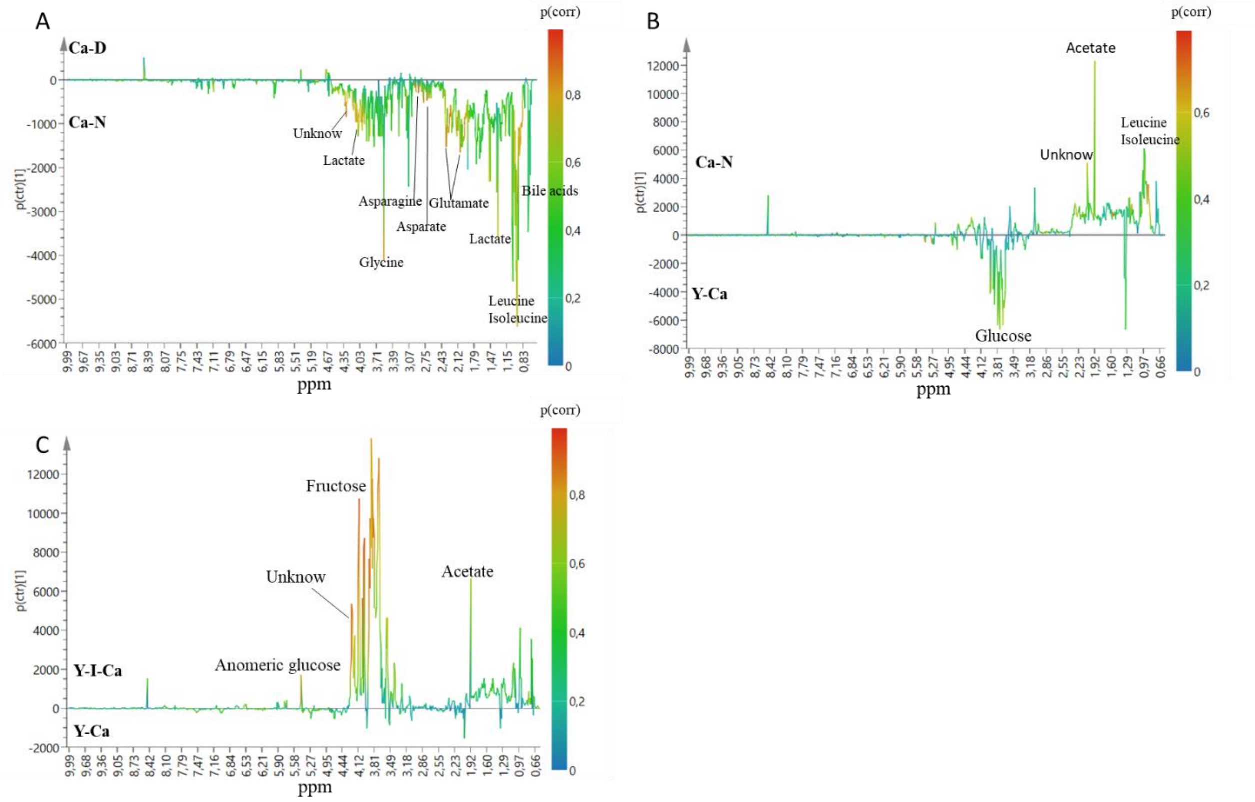

Figure S3. S-line plots of OPLS-DA to visualize the signal differences in the NMR spectra between (A) Ca-D vs. Ca-N (R^2=0.98, Q^2 =0.87), (B) Ca-N vs. Y-Ca (R^2=0.79, Q^2 =0.68), and (C) Y-Ca vs. Y-I-Ca (R^2=0.90, Q^2 =0.75) in jejunal content.

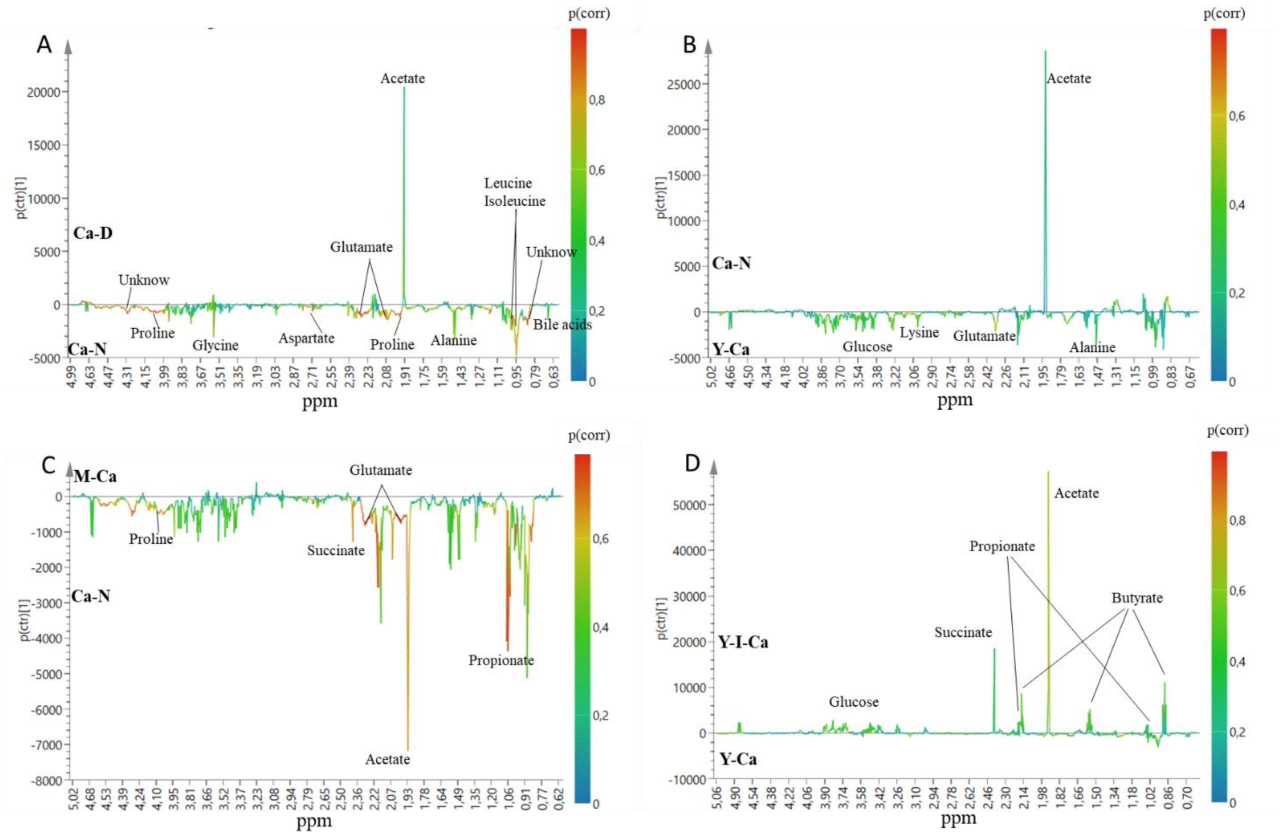

Figure S4. S-line plots of OPLS-DA to visualize the signal differences in the NMR spectra (0.6 ppm-10.00 ppm) between (A) Ca-D vs. Ca-N (R^2=0.91, Q^2=0.83), (B) Ca-N vs. Y-Ca (R^2=0.98,Q^2=0.72), (C) Ca-N vs. M-Ca (R^2=0.97, Q^2=0.54), and (D) Y-Ca vs. Y-I-Ca (R^2=0.98, Q^2=0.86) in cecal content.

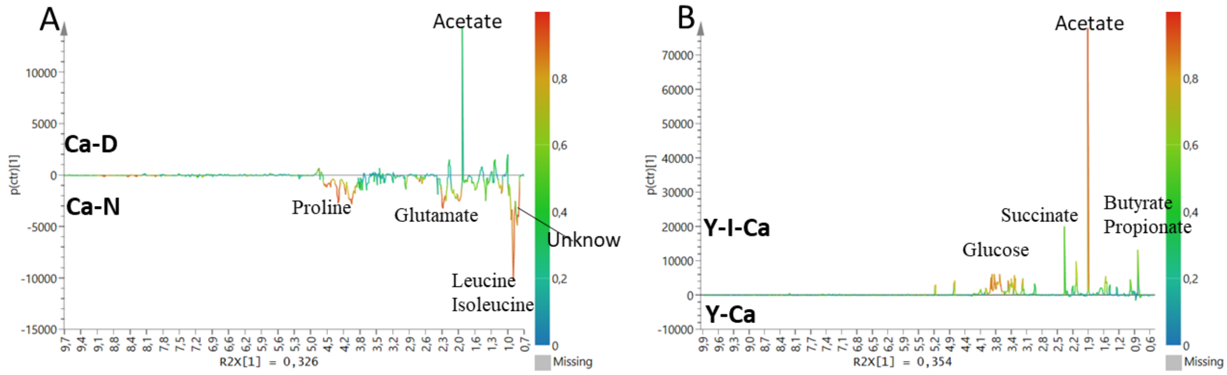

Figure S5. S-line plots of OPLS-DA to visualize the signal differences in the NMR spectra between (A) Ca-D vs. Ca-N (R^2=0.93, Q^2 =0.83) and (B) Y-Ca vs. Y-I-Ca (R^2=0.84, Q^2=0.56) in colon content.

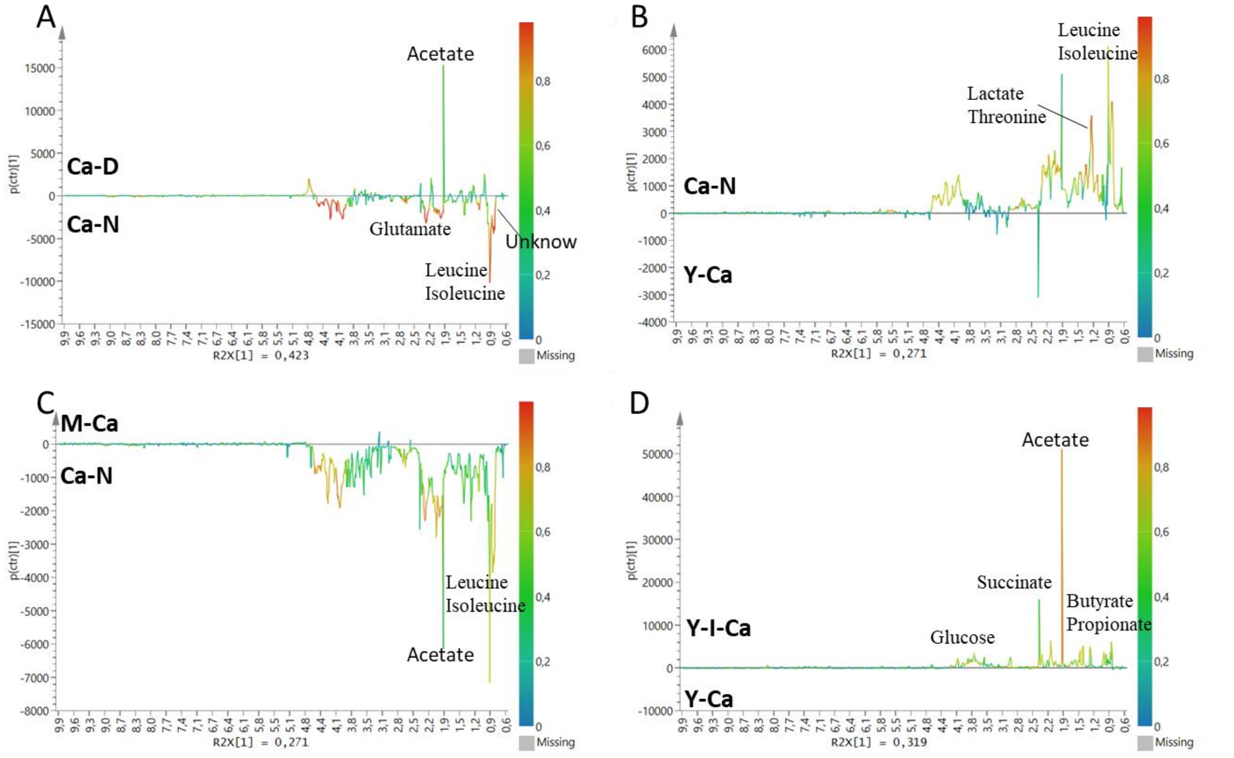

Figure S6. S-line plots of OPLS-DA to visualize the signal differences in the NMR spectra between (A) Ca-D vs. Ca-N (R^2=0.94, Q^2=0.90), (B) Ca-N vs. Y-Ca (R^2=0.94, Q^2=0.76), (C) Ca-N vs M-Ca (R^2=89, Q^2=0.70), and (D) Y-Ca vs Y-I-Ca (R^2=0.97, Q^2=0.81) in feces.

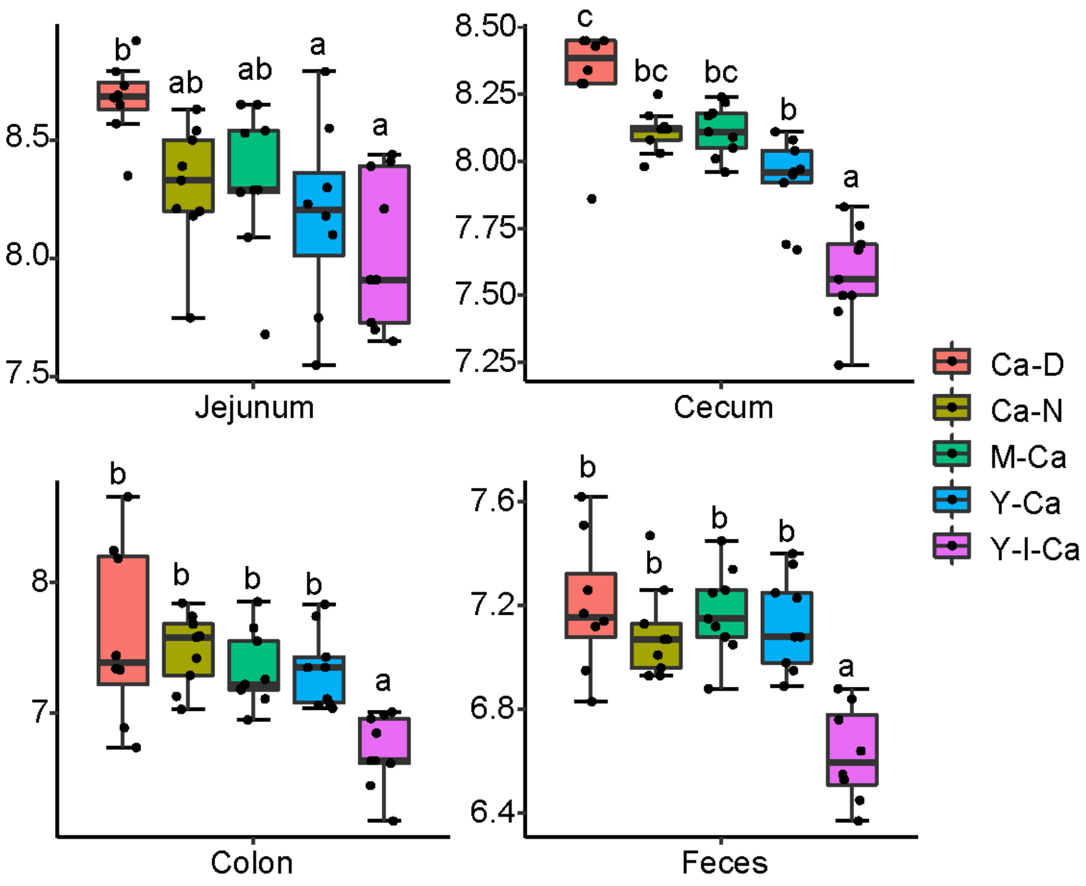

Figure S7. The pH of jejunal content, cecal content, colon content, and feces. Different low-case letters indicate significant differences between experimental groups ($p < 0.05$)

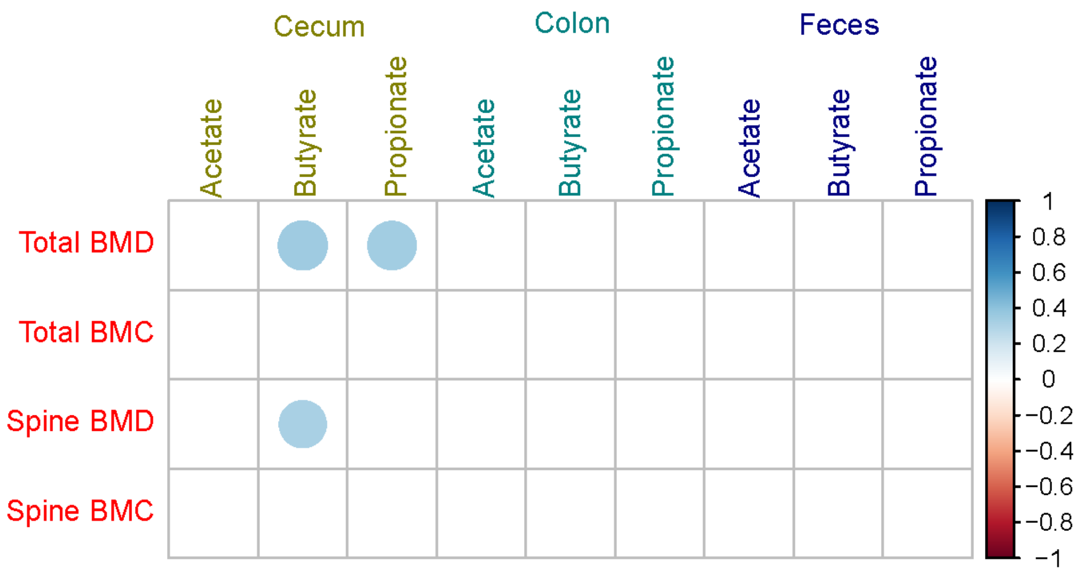

Figure S8. Pearson correlations between the concentrations of SCFAs and bone parameters. The symbol circle represents significant correlations (p<0.05) between parameters, and the corresponding color shows the value of correlation coefficients.

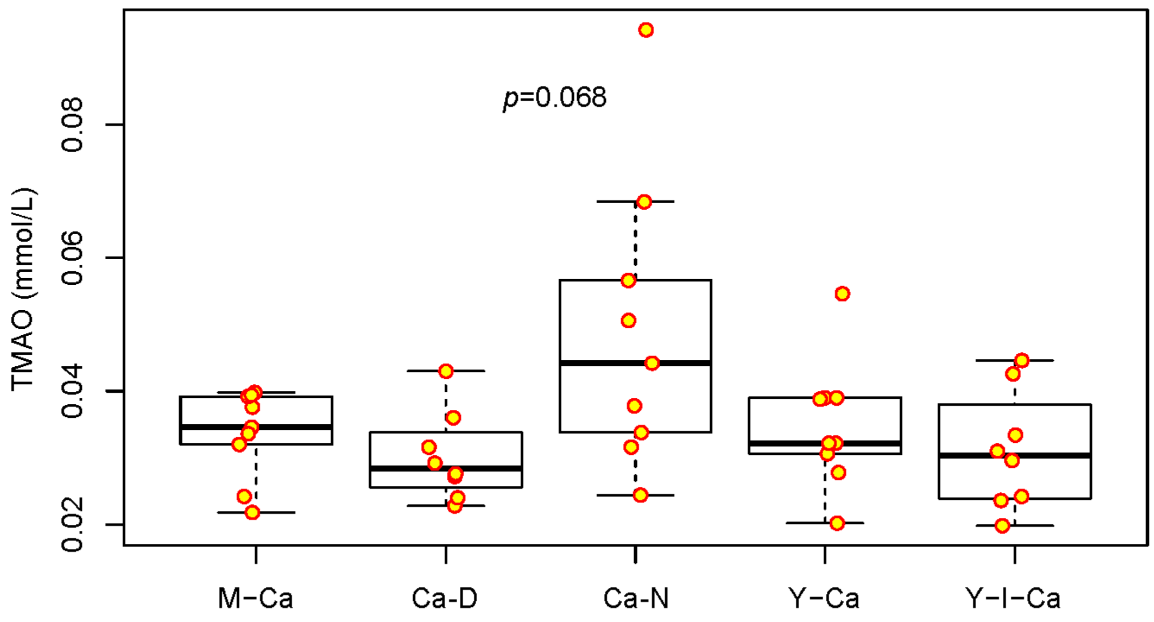

Figure S9. The serum concentration of trimethylamine N-oxide (TMAO) measured by NMR.

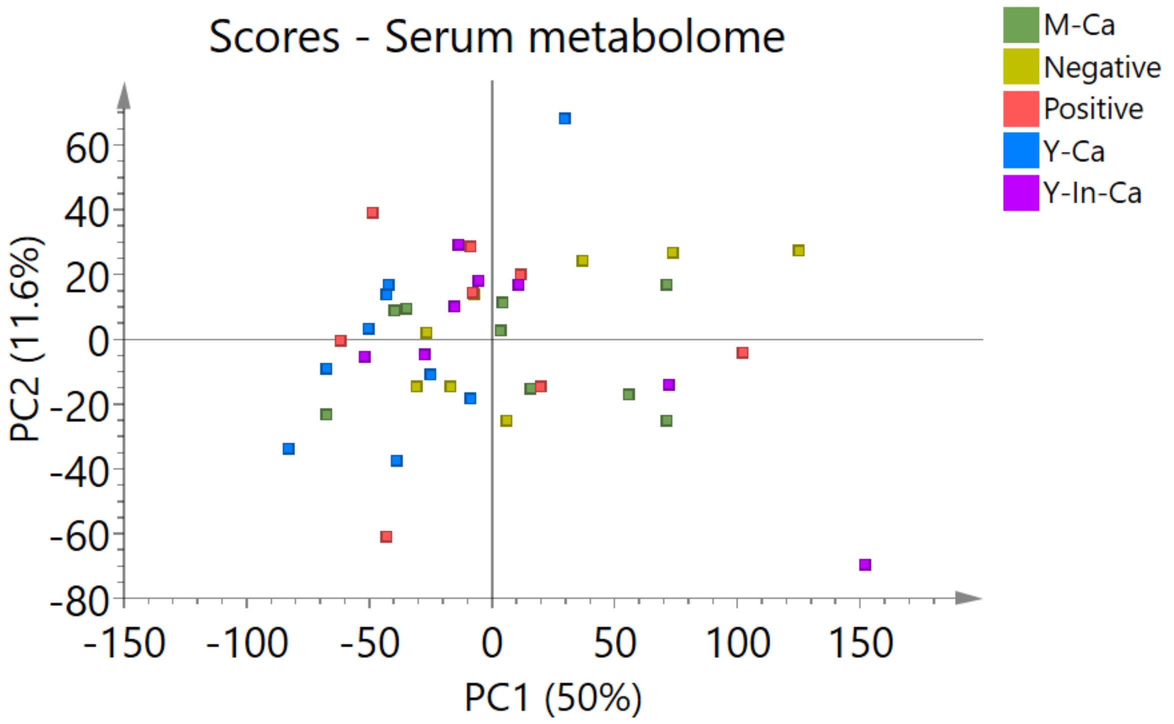

Figure S10. Scores plots of PCA for all NMR metabolite profiles of serum.

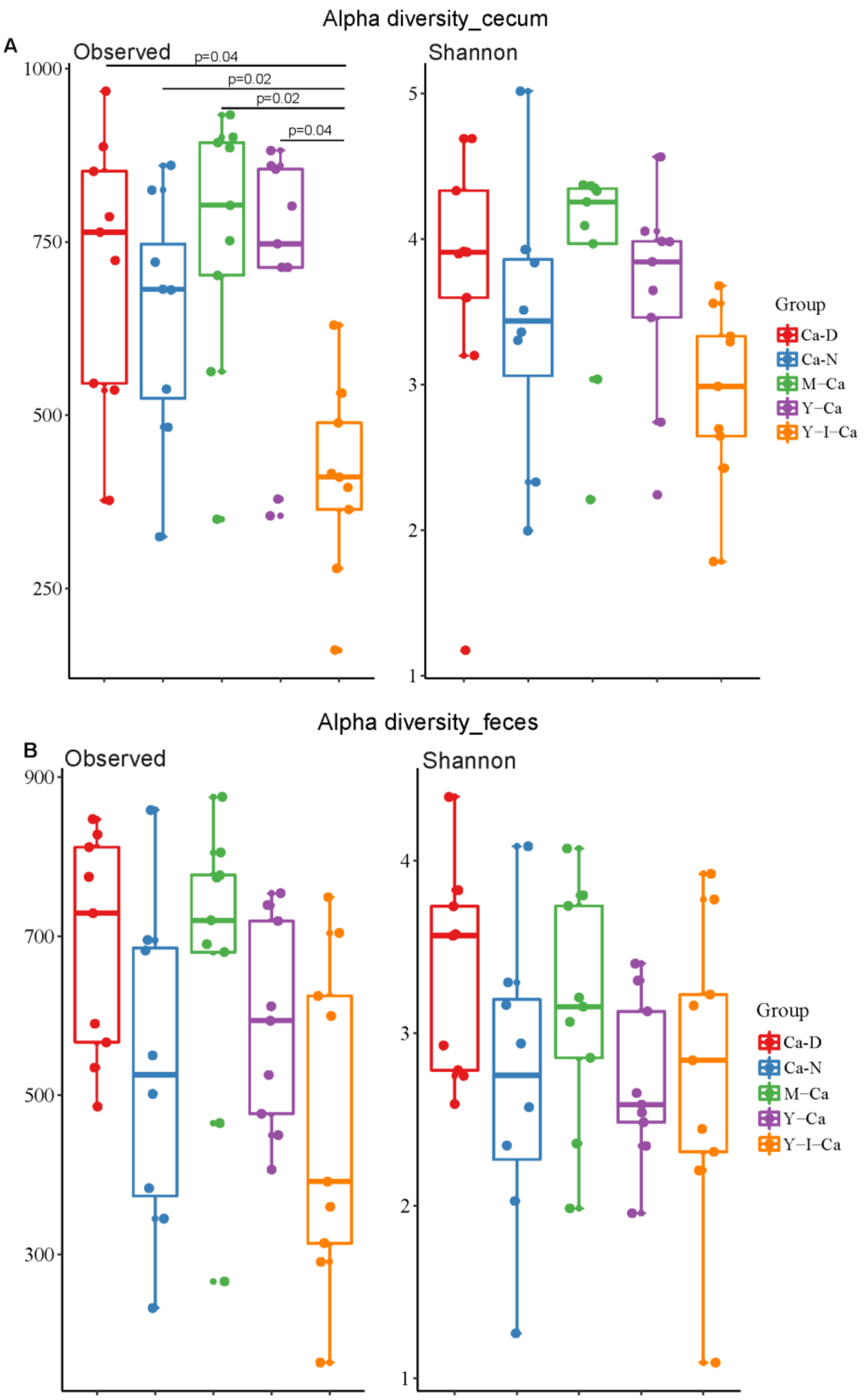

Figure S11. The observed index and Shannon index of 16S rRNA sequencing-based alpha diversity in cecal content (A) and feces (B).

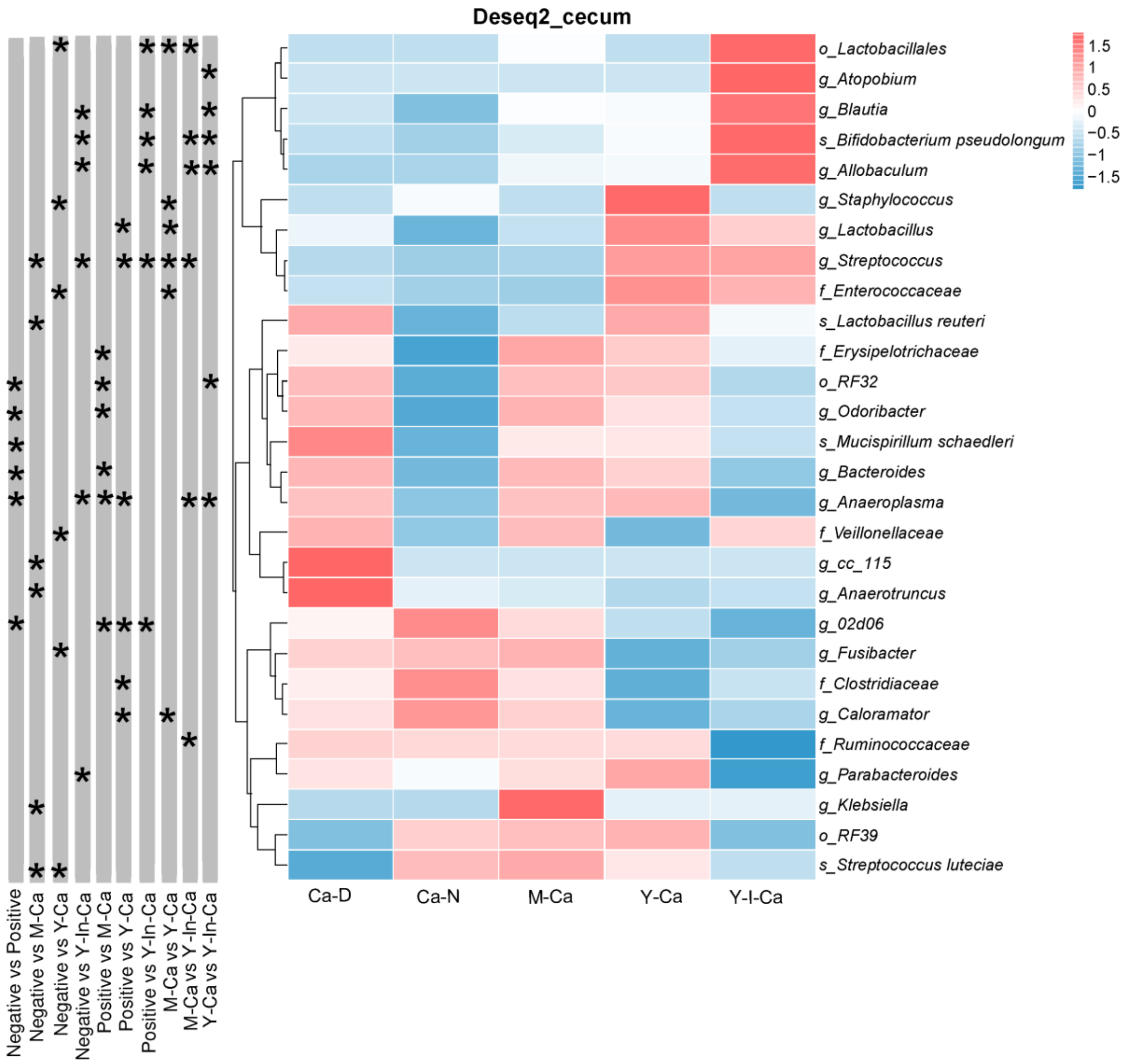

Figure S12. Heatmap of the relative abundance of bacteria with indication of significant differences among groups in cecal content Symbol * represents $p<0.05$ between the compared two groups.

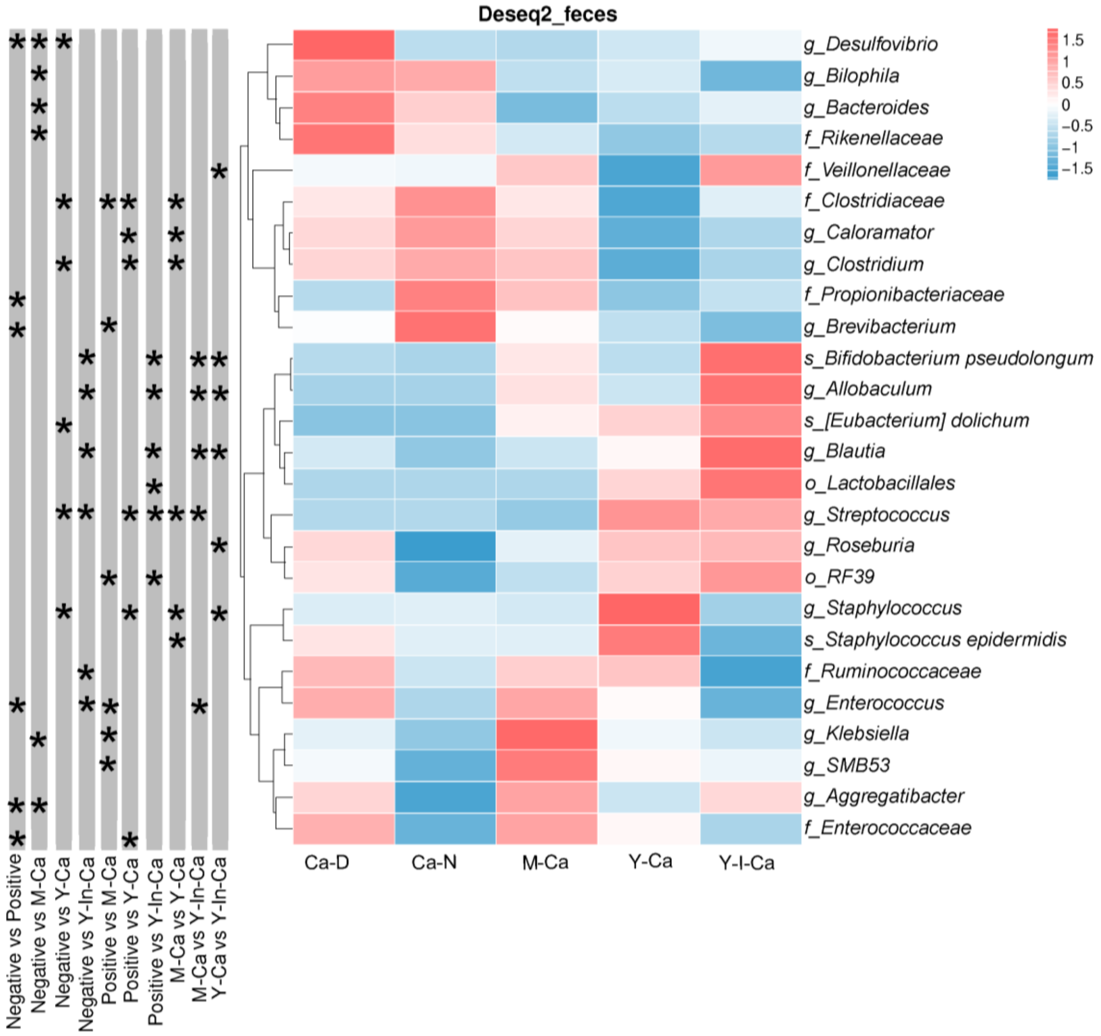

Figure S13. Heatmap of the relative abundance of bacteria with indication of significant differences among groups in feces. Symbol * represents $p<0.05$ between the compared two groups.

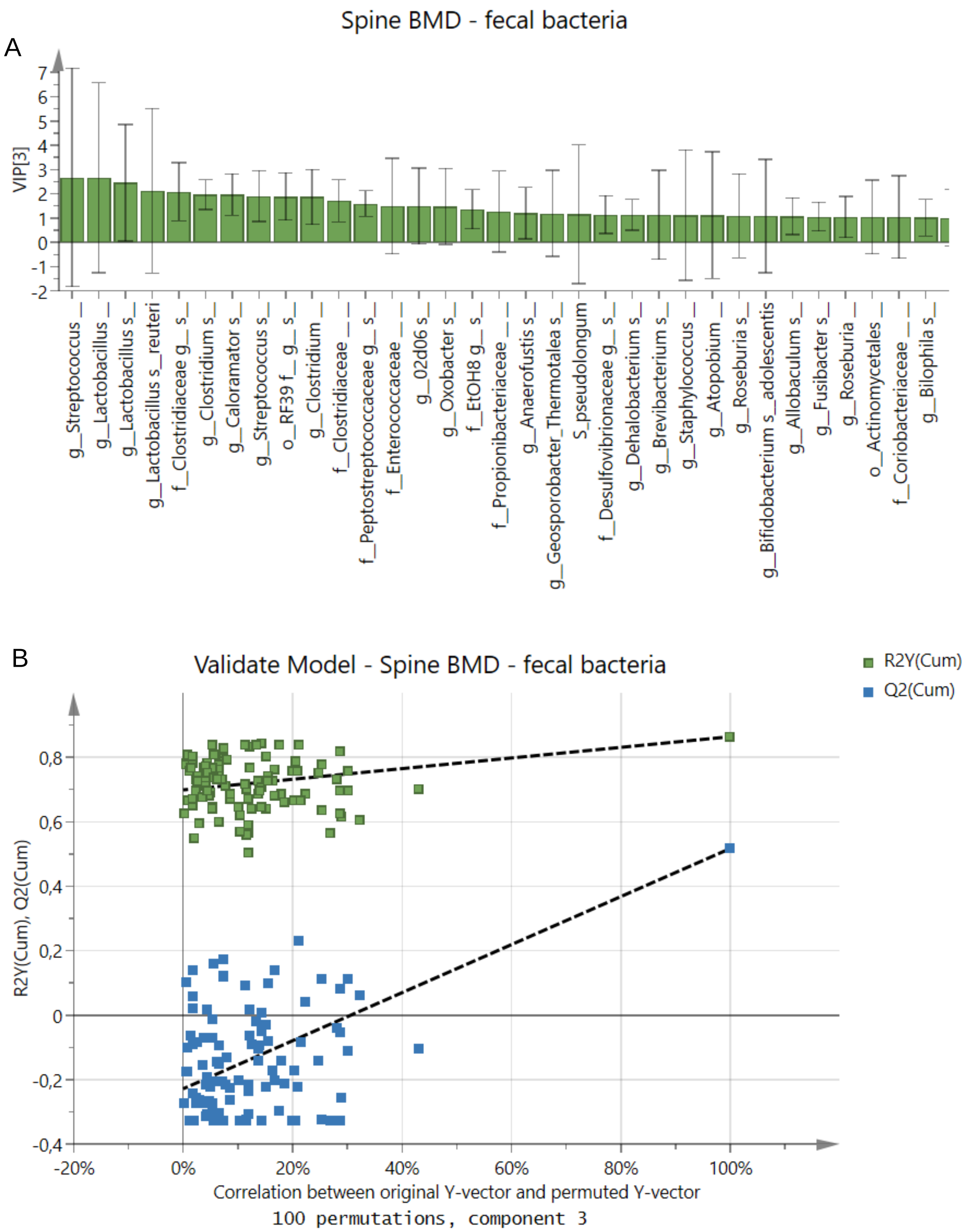

Figure S14. The variable important projection (VIP) plot (A) and Permutation plot (B) for the PLS model (R^2=0.86, Q^2=0.54) between fecal microbiota compositions and the spine BMD (n=44). VIP plot can reveal important variables correlated to Y (spine BMD). The Permutation plot indicates that this PLS model is robust

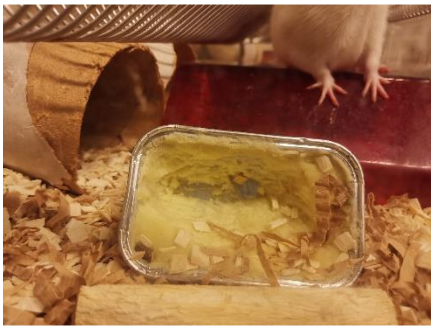

Figure S15. A food paste left over after 24 hours provided to rats
